# Cytosine methylase and hydroxymethylase activity in mammalian mitochondria

**DOI:** 10.3389/fcell.2025.1677402

**Published:** 2025-10-15

**Authors:** Lisa S. Shock, Prashant V. Thakkar, Jason M. Robinson, Shirley M. Taylor

**Affiliations:** ^1^ Department of Microbiology and Immunology, Virginia Commonwealth University School of Medicine, Richmond, VA, United States; ^2^ The Massey Comprehensive Cancer Center, Virginia Commonwealth University School of Medicine, Richmond, VA, United States

**Keywords:** mitochondrial DNA (mtDNA), epigenetics, transcription, DNA replication, DNA methylation, DNA demethylation, DNA methyltransferase

## Abstract

**Introduction:**

Mitochondria are integral components of eukaryotic cells, functioning as energy powerhouses and key mediators of diverse metabolic and signaling cascades. As endosymbiotic remnants, these unique organelles retain and express their own DNA. Mitochondrial DNA (mtDNA) is packaged into DNA-protein complexes called nucleoids, and is also subject to epigenetic modification. We identified a mitochondrial isoform of DNA methyltransferase 1 (mtDNMT1) that binds to mtDNA in critical control regions; however, its enzymatic activity remained unexplored.

**Results:**

Here, we show that endogenously-tagged mtDNMT1 purified from mitochondria exhibits time- and concentration-dependent CpG-specific DNA methyltransferase activity, but it is not working alone: DNMT3b cooperates with mtDNMT1 to methylate mtDNA and regulate mitochondrial transcription. In addition, we detect ten-eleven translocase (TET)-like hydroxymethylase activity in mitochondria, demonstrating that mechanisms for both writing and erasing 5-methylcytosine marks are functional in this organelle. CRISPR/Cas9-mediated inactivation of mtDNMT1 and/or DNMT3b activity resulted in a stepwise decrease in mitochondrial methylation across the heavy and light strand promoters of mtDNA, with a significant reduction in transcription of several mtDNA-encoded OXPHOS genes. Interestingly, the effects of mtDNA methylation on mitochondrial transcription are diametrically opposed to the role of promoter methylation in the nucleus, suggesting a novel mode of gene regulation in mitochondria. Cells lacking mtDNMT1 and/or DNMT3b also exhibited a modest reduction in mtDNA content, suggesting that methylation impacts both mtDNA transcription and replication.

**Discussion:**

These observations implicate mtDNA methylation in the fine-tuning of mitochondrial function and suggest a role for aberrant mitochondrial methylase activity in disease.

## 1 Introduction

Mammalian mitochondrial DNA (mtDNA) exists as a 16.5 kb double-stranded, circular molecule, present in ∼2–10 copies per mitochondrion and roughly 100–1,000 copies per somatic cell (Garcia et al., 2017; Gilkerson et al., 2013). The mitochondrial genome encodes a total of 37 genes, encoding 2 rRNAs, 22 tRNAs and 13 proteins essential for proper assembly and function of the respiratory chain complexes (Gustafsson et al., 2016). The vast majority of mitochondrial proteins are encoded in the nucleus, synthesized on cytosolic ribosomes and either co- or post-translationally transported into mitochondria (Wiedemann and Pfanner, 2017). Thus, the bioenergetics of the cell as a whole relies on the precise coordination of both nuclear and mitochondrial transcriptional and translational programs.

Mitochondria are devoid of histones, so packaging of mtDNA is achieved through the formation of nucleoprotein complexes called nucleoids. These macromolecular assemblies provide an efficient segregating unit, ensuring proper inheritance and distribution of mtDNA throughout the cell, and coupling the partitioning of mtDNA with metabolism (Gilkerson et al., 2013; Bogenhagen et al., 2008; Chen and Butow, 2005). By far the most abundant protein in mitochondrial nucleoids is the mitochondrial transcription factor A (TFAM), which plays an interesting dual-functional role as both a structural/packaging protein and an integral member of the transcription initiation complex (Bogenhagen et al., 2008; Ngo et al., 2014).

Transcription of the mitochondrial genome is executed by a three-component transcription initiation complex, comprised of a single-subunit DNA-directed RNA polymerase mitochondrial (POLRMT), and its auxiliary factors TFAM and mitochondrial transcription factor B2 (TFB2M) (Ramachandran et al., 2017; Morozov et al., 2015; Hillen et al., 2018). Transcription initiates from two closely opposed promoters: the light-strand promoter (LSP) and the heavy-strand promoter (HSP), and proceeds in a bidirectional fashion, yielding polycistronic transcripts that are further processed into mature mRNAs, rRNAs and tRNAs (Gustafsson et al., 2016; Morozov et al., 2015; Hillen et al., 2018). A termination factor (mTERF) binding site immediately downstream of the rRNA genes allows the high-level production of rRNA transcripts required for protein synthesis (Hyvarinen et al., 2007). Notably, in addition to generating mRNA, tRNA and rRNA, mtRNAP also synthesizes the RNA primer required for mtDNA replication (Wanrooij et al., 2008). In this way, the regulation of transcription initiation is inextricably linked with mtDNA maintenance and organelle biogenesis.

It is well-established that nuclear DNA is subject to various forms of epigenetic regulation, including DNA methylation, histone modification and nucleosome remodeling. The coordinated effects of these modifications result in an “epigenetic code,” which affects local chromatin structure and ultimately, gene expression. DNA methylation arises through the addition of a methyl group to the C5 position of a cytosine residue and occurs almost exclusively in a CpG dinucleotide context. Dense clusters of CpG dinucleotides (termed “CpG islands”), are found in the promoter regions of ∼70% of all genes (Deaton and Bird, 2011). For the majority of these genes, promoter activity is repressed when methylated, and thus, DNA methylation is typically inversely correlated with gene expression. Cytosine methylation is catalyzed by a family of DNA methyltransferase (DNMT) enzymes: the *de novo* methylases, DNMT3a and DNMT3b are responsible for establishing the methylation pattern on unmethylated DNA; this pattern is then faithfully propagated by the maintenance methyltransferase, DNMT1.

While stable and heritable, DNA methylation is also dynamic and reversible. 5-methylcytosine (5mC) can be removed either by passive dilution, when methylation is not maintained through subsequent rounds of DNA replication, or by active demethylation, catalyzed by the ten-eleven translocase (TET) family of dioxygenase enzymes. In active DNA demethylation, 5mC is converted through iterative oxidation steps to 5-hydroxymethylcytosine (5hmC), then 5-formylcytosine (5fC), and 5-carboxycytosine (5caC) (Tahiliani et al., 2009; Ito et al., 2011). Either 5fC or 5caC can be recognized and removed by thymine DNA-glycosylase (TDG) activity during base excision repair, ultimately replacing the modified base with an unmodified cytosine (Shi et al., 2017). Turnover of both 5 fC and 5caC are relatively rapid, contributing to their low steady-state abundance in various cells and tissues (Ito et al., 2011). However, 5hmC is present at high levels in the central nervous system, embryonic stem cells (ESCs) and in some somatic tissues; both its stability and enrichment at gene regulatory regions (promoters, enhancers, and transcription-factor binding sites) suggest that 5hmC has regulatory functions of its own (Tahiliani et al., 2009; Ito et al., 2011; Shi et al., 2017). Three TET proteins (TET1, TET2 and TET3) possess methylcytosine oxidase activity and are capable of converting 5mC to 5hmC/5fC/5caC, and each enzyme has a unique cell- and tissue-specific expression pattern owing to their distinct roles in cellular and developmental gene expression programs (Ito et al., 2011; Shi et al., 2017; Huang et al., 2014).

Whether mtDNA contains functionally significant levels of epigenetic modification has been debated for decades (Nass, 1973; Shmookler Reis and Goldstein, 1983; Pollack et al., 1984). Previous work from our lab revealed the presence of not only 5mC but also 5hmC residues in mtDNA, suggesting that the potential impact of cytosine methylation in mitochondria had been underestimated (Shock et al., 2011). Since then, several studies have reported the detection of one or more DNA methyltransferase (Shock et al., 2011; Saini et al., 2017; Dostal and Churchill, 2019; Maresca et al., 2020; Hao et al., 2020; Wong et al., 2013; Bellizzi et al., 2013), and TET hydroxymethylase (Bellizzi et al., 2013; Dzitoyeva et al., 2012; Ji et al., 2018), proteins localized to mitochondria, however the catalytic activity of these enzymes remained untested. We approached these questions with the goal of elucidating the function of these enzymes in mitochondria: determining whether they were indeed capable of writing and erasing mtDNA methylation, and investigating the impact of their enzymatic activity on mitochondrial physiology.

Herein we report a detailed mechanistic study of the functional consequences of altering cytosine methylation levels in mtDNA. We demonstrate that both mtDNMT1 and DNMT3b are catalytically active within mitochondria, and altered expression of the mtDNMTs leads to changes in 5mC levels across the D-loop control region of mtDNA with resultant changes in mitochondrial transcription and mtDNA copy number. We also provide the first report of TET-like hydroxymethylase activity, capable of converting 5mC to 5hmC, originating from within mitochondria. Surprisingly, our data shows that decreasing 5mC levels in mtDNA serves to decrease mitochondrial transcription, which is contradictory to the role of promoter DNA methylation in the nucleus. Thus, our findings indicate that mtDNA methylation impacts mitochondrial transcription in a fashion that is distinctly different from epigenetic regulation in the nucleus, suggesting a novel functional significance for mitochondrial epigenetics that remains to be fully explored.

## 2 Results

### 2.1 mtDNMT1 and DNMT3b, but not DNMT3a, are catalytically active in mitochondria

**FIGURE 1:**
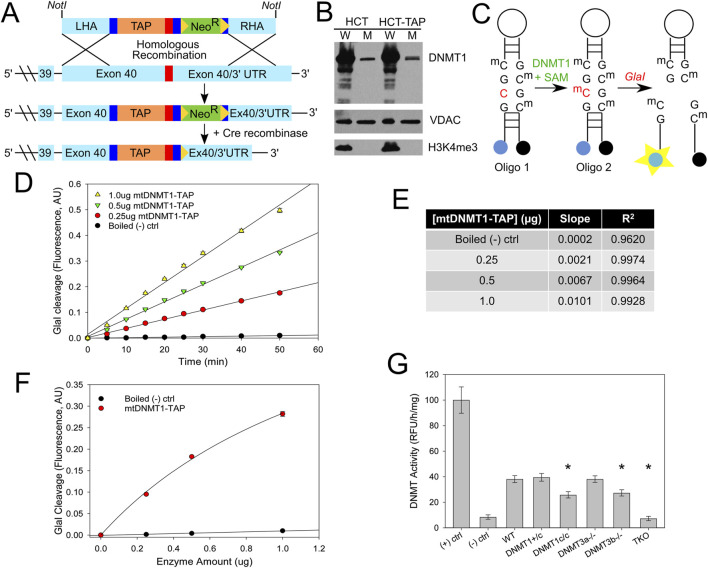
mtDNMT1 and DNMT3b are catalytically active in mitochondria. **(A)** The fusion PCR strategy used to generate the HCT116 TAP-tagged DNMT1 cell line. LHA, left homology arm; RHA, right homology arm; TAP, tandem affinity purification tag; Neo^R^, neomycin resistance cassette. **(B)** Immunoblots showing DNMT1-TAP localizes properly to the mitochondrial compartment and is expressed at near-endogenous levels. VDAC, voltage-dependent anion channel (mitochondrial loading control); H3K4me^3^, histone 3 lysine 4 tri-methylation (nuclear contamination control). **(C)** Schematic of the CpG-specific methyltransferase assay used in **(D–F)**. **(D)** DNMT1-TAP purified from mitochondria exhibits CpG-specific methyltransferase activity that is linear with respect to time. **(E)** Slope and *R* ^2^ linear correlation values for CpG methyltransferase activity of mtDNMT1-TAP, plotted in **(D) (F)** mtDNMT1-TAP exhibits classic Michaelis-Menten kinetics when methyltransferase activity is plotted over enzyme concentration. **(G)** DNMT1 and DNMT3b, but not DNMT3a, contribute to total methylation activity in mitochondria isolated from murine ES cells. (+) ctrl, positive M. *Sss*I CpG methylase control; (-) ctrl, Boiled WT negative control; DNMT1+/c, heterozygous catalytic knockout of DNMT1 (1 allele); DNMT1c/c, homozygous catalytic knockout of DNMT1 (both alleles); DNMT3a−/−, DNMT3a knockout; DNMT3b−/−, DNMT3b knockout; TKO, triple knockout, lacking all three DNMTs. Experiments consisted of two biological replicates, each with at least three technical replicates. Error bars represent the standard deviation about the mean. Statistical significance was determined by one-way ANOVA with Tukey’s post-hoc test, *p < 0. 05.

We inserted a tandem affinity purification (TAP) tag at the C-terminal endogenous locus of a single DNMT1 allele in HCT116 cells by AAV-mediated homologous recombination (Rigaut et al., 1999; Kohli et al., 2004). The DNMT1-TAP targeting construct was generated by a series of fusion PCR reactions, linking the TAP-tag and neomycin resistant cassette, which were then flanked by regions of homology to the C-terminus of human DNMT1 to facilitate recombination (Figure 1A). HCT116-DNMT1-TAP (HCT-TAP) cells were fractionated into whole cell and mitochondrial fractions, and immunoblots show that DNMT1-TAP localized properly to the mitochondrial compartment (Figure 1B). Mitochondrial DNA immunoprecipitation (mtIP) analyses detected that DNMT1-TAP directly bound mtDNA with a frequency that is proportional to CpG density (Shock et al., 2011), and partitions with the insoluble mtDNA nucleoid (Supplementary Figure S1), proving its presence within the mitochondrial matrix. We adapted the sensitive fluorescence-based *Gla*I cleavage assay developed by the Roach laboratory (Wood et al., 2010) to measure the methyltransferase activity of mtDNMT1-TAP purified from the mitochondrial fraction of cells (Figures 1C–F). The assay relies on a unique break light oligonucleotide, labeled at its 5′ end with a FAM fluorophore and at its 3′ end with a dabcyl quencher. The oligonucleotide forms a hairpin, which locks the fluorophore and quencher in close proximity, preventing fluorescence. Within the stem of the hairpin are four symmetric CpG sites, which can be either hemi-methylated (Oligo 1, Figure 1C) or fully methylated (Oligo 2, Figure 1C). When fully tetra-methylated, the oligonucleotide becomes the optimal substrate for the methylation-sensitive restriction enzyme, *Gla*I. Cleavage by *Gla*I releases the fluorophore from the quencher, producing an increase in fluorescence that is directly proportional to the degree of CpG methylation. Mitochondria were isolated from HCT-TAP cells, the intact mitochondrial pellet was digested with trypsin to proteolyze any contaminating proteins bound to the outer mitochondrial membrane surface. Upon lysis of mitochondria, mtDNMT1-TAP was affinity-purified as described (Rigaut et al., 1999). Increasing amounts of purified mtDNMT1-TAP were incubated with 240 nM Oligo 1, 1 mM S-adenosylmethionine, and 0.8U *Gla*I to measure its methylation activity over time. Purified mtDNMT1-TAP exhibited CpG-specific DNA methyltransferase activity that was linear across time and obeyed classical Michaelis-Menten kinetics when plotted versus enzyme concentration (Figures 1D–F). This activity was easily detectable above boiled mtDNMT1-TAP (negative) controls, and its resistance to trypsin digestion prior to mitochondrial lysis verifies its origin from within the mitochondrial compartment.

In our previous work (Shock et al., 2011), we were unable to detect the presence of either DNMT3a or DNMT3b in mitochondria by immunoblot. Those observations, combined with the lack of any conserved sequences bearing resemblance to a mitochondrial targeting peptide, led us to conclude that the *de novo* methyltransferases did not translocate to mitochondria or contribute to the mtDNA methylation profile. However, because other groups have more recently reported the presence of DNMT3a and/or DNMT3b in mitochondria (Wong et al., 2013; Bellizzi et al., 2013), we revisited the question of whether either of the *de novo* enzymes might also cooperate with mtDNMT1 to methylate mtDNA. We obtained a family of embryonic stem (ES) cell lines carrying catalytic deletions in each of the DNMTs (Okano et al., 1999). We isolated highly purified mitochondrial fractions from each cell line and measured the total DNA methyltransferase activity using a sensitive, fluorometric ELISA-like assay. Briefly, 96-well plates pre-coated with universal unmethylated DNA substrate were incubated with purified mitochondrial extracts; active DNMTs within those mitochondrial extracts transferred methyl groups to unmodified cytosines. The level of methylated DNA, which is proportional to enzyme activity, was detected through binding by a fluorescent-labeled anti-5mC antibody and measured as fluorescence intensity. Immunoblots demonstrated the purity of mitochondrial extracts (Supplementary Figure S2). Cells lacking both alleles of DNMT1 (DNMT1c/c) or DNMT3b displayed appreciable decreases in mitochondrial DNMT activity relative to WT controls (Figure 1G). This suggests a functional cooperation between mtDNMT1 and DNMT3b, similar to their reciprocal and compensatory relationship in the nucleus (Rhee et al., 2002). Mitochondrial methylation in cells lacking all three DNMTs (triple knockout, TKO) was at baseline/background levels. Notably, genetic loss of DNMT3a alone showed no effect on total DNMT activity originating from the mitochondrial compartment, suggesting that DNMT3a is not involved in methylating mtDNA (Figure 1G). However, we are relying on the single KO of each DNMT individually to substantiate their involvement and drawing inference from the TKO line that only DNMT1 and DNMT3b cooperate in mitochondria. Without the full complement of double knockout cells (DNMT1/3b, DNMT1/3a, DNMT3a/3b) from mice that were not generated (Okano et al., 1999), we cannot definitively discount any contribution by DNMT3a.

### 2.2 TET-like DNA hydroxymethylase activity and TET2 are detected in mitochondria

**FIGURE 2:**
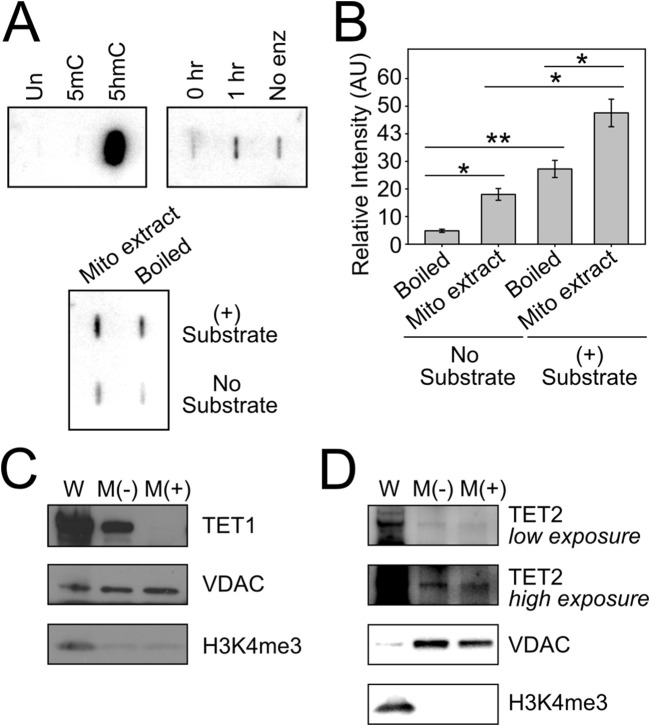
Tet hydroxymethylase activity and TET2 in mitochondria. **(A)** *In vitro* Tet enzyme assay detects hydroxymethylase activity in mitochondria. **(B)** Densitometry of slot blots in **(A) (C)** Immunoblot analysis of whole cell and purified mitochondria from HCT116 cells show that TET1 localizes to the outer mitochondrial membrane, and is not resistant to enzymatic digestion of the intact mitochondrial pellet. M(-), untreated mitochondria; M(+), trypsin-digested mitochondria; VDAC, mitochondrial loading control; H3K4me^3^, nuclear contamination control. **(D)** Immunoblot analysis of whole cell and purified mitochondria demonstrate that TET2 is protected from enzymatic digestion of the mitochondrial pellet, suggesting its localization within mitochondria. Error bars represent the standard deviation about the mean for two independent experiments, each with two technical replicates. Statistical significance was determined by two-tailed, two-sample Student’s t-test, *p < 0.05, **p < 0.01.

Having confirmed the presence of DNA methyltransferase activity in mitochondria, we sought to define the mechanism(s) involved in the generation of 5hmC in mtDNA, with a focus on TET-mediated oxidation of 5mC. We used an *in vitro* slot blot assay to detect TET enzyme activity within purified mitochondria. Briefly, denatured substrate DNA (100 ng of methylated human APC promoter sequence) was incubated with purified mitochondrial lysate, which served as the source of potential TET enzyme activity (Liu et al., 2016). TET enzyme reactions were incubated for 0–1 h at 37 °C and terminated by quenching on ice. The reaction products, containing converted 5hmC residues, were slotted onto an absorbent membrane and probed with a modification-specific antibody for 5hmC. The resultant 5hmC signal was detected with chemiluminescence and quantified by densitometry. For positive control reactions (Figure 2A, top panels), 0.4 ng of unmethylated (Un), methylated (5mC) and hydroxymethylated (5hmC) DNA was spotted onto a membrane to determine the specificity of our 5hmC antibody, which we validated to detect as little as one 5hmC in 200 cytosine residues. We used recombinant TET1 C-terminal domain (TET1-CTD) as a positive control for enzyme activity. For negative controls, mitochondrial lysate was boiled for 10 min to abolish any TET enzyme activity prior to incubation with substrate DNA, and separate reactions were performed without substrate DNA. Total intact mitochondria were isolated from WT HCT116 cells and subjected to trypsin digestion to eliminate any contaminating cytosolic or nuclear proteins bound to the outer mitochondrial membrane. Mitochondrial lysates were deemed to be free from nuclear contamination by Western blot (Figures 2C,D). As shown in Figure 2A (bottom panels) and quantified in Figure 2B, we detected TET enzyme activity in trypsin-treated mitochondrial lysates (Mito extract, (+) Substrate), above the levels in no-substrate and boiled-enzyme controls. Background signal in the Boiled (-) controls represents endogenous 5hmC in mtDNA, which is also present in mitochondrial lysates, and background signal in the No Substrate lanes is indicative of enzyme-dependent catalysis of endogenous 5mC in mtDNA. This represents the first report of hydroxymethylase activity originating from within the mitochondrial compartment, and provides a direct mechanism for the generation of 5hmC in mtDNA.

A few independent groups have reported that either TET1 and/or TET2 localize to mitochondria (Bellizzi et al., 2013; Dzitoyeva et al., 2012; Ji et al., 2018). We explored whether one of these hydroxymethylase enzymes may be responsible for the conversion of 5mC to 5hmC that we detect *in vitro*. Purified mitochondrial extracts were digested with trypsin, separated by SDS-PAGE and probed with an antibody directed against either TET1 or TET2. We detected TET1 immunoreactivity in Mock/untreated mitochondrial extracts (M(-)), however, this signal was sensitive to trypsin digestion of intact mitochondria (M(+)), suggesting that TET1 is bound only to the outer mitochondrial membrane and does not gain access to inner mitochondrial compartments (Figure 2C). Notably, however, we detected TET2 immunoreactivity in both Mock and trypsin-treated mitochondrial extracts, supporting the presence of TET2 in this organelle (Figure 2D).

Considering the *in vitro* evidence that DNMT1 is capable of catalyzing direct addition of a hydroxymethyl group onto cytosine to form 5hmC (Liutkeviciute et al., 2009), and the known abundance of reactive oxidation intermediates in mitochondria as by-products of respiration, we asked whether mtDNMT1 might exhibit some alternative oxidase activity in mitochondria. Upon genetic deletion of DNMT1 in murine ES cells, methyl-DNA immunoprecipitation (MeDIP) analysis detected the expected reduction in 5mC levels across 12S and 16S rRNA regions of mtDNA, dropping to ∼35% of WT levels in DNMT1^−/−^ ES cells (Supplementary Figure S4A). Notably, hydroxy-MeDIP performed on the same cells displayed a stepwise *increase* in 5hmC levels upon loss of one or both allele(s) of DNMT1 (Supplementary Figure S4B). While somewhat surprising, these findings are in agreement with work from others (Veto et al., 2018; Chowdhury et al., 2015) which detected increased 5hmC levels upon treatment of cells with the DNMT inhibitor decitabine, and lend further support to the conclusion that mtDNMT1 does not generate 5hmC in mitochondria. Combined, these data suggest that TET2 is likely responsible for the hydroxymethylase activity detected in our *in vitro* enzyme assays (Figures 2A,B), and functions to convert 5mC to 5hmC residues in mtDNA.

### 2.3 Overexpression of mtDNMT1 increases cytosine methylation and transcription of the mitochondrial heavy strand

**FIGURE 3:**
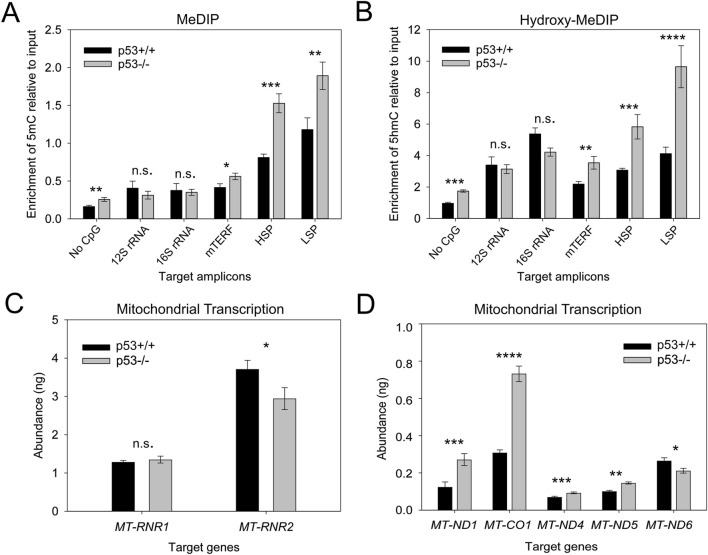
Upregulation of mtDNMT1/loss of p53 increases mitochondrial 5mC and 5hmC levels and exerts gene-specific effects on transcription. **(A)** MeDIP analysis shows increased 5mC enrichment in the HSP and LSP regions of mtDNA upon mtDNMT1 upregulation/loss of p53. **(B)** Hydroxy-MeDIP analysis shows similar increases in 5hmC levels upon mtDNMT1 upregulation/loss of p53. **(C,D)** Upregulation of mtDNMT1/loss of p53 induces gene-specific effects on mitochondrial transcription. mTERF, mitochondrial termination factor site; HSP, heavy-strand promoter; LSP, light strand promoter; ND4c, light strand complementary to ND4; ND5c, light strand complementary to ND5. Experiments consisted of at least two biological replicates, each with at least three technical replicates. Statistical significance was determined by two-tailed, two-sample Student’s t-test, *p < 0.05, **p < 0.01, ***p < 0.005, ****p <0 .0005, n. s., not significant.

We previously showed that expression of mtDNMT1 is significantly upregulated upon loss of p53 (Shock et al., 2011). Here, we asked how the change in mtDNMT1 expression impacts mtDNA methylation levels at critical mtDNA control regions. Genomic DNA purified from HCT116 p53^+/+^ and p53^−/−^ cells, was examined by MeDIP and hydroxy-MeDIP assays. Upon genetic loss of p53/upregulation of mtDNMT1, we observed approximate 2-fold increases in the abundance of 5mC and 5hmC residues across both the heavy- and light-strand promoters (HSP and LSP, respectively) (Figures 3A,B). The mTERF binding site, responsible for coordinating high-level production of rRNA transcripts, also showed increased levels of 5mC and 5hmC upon upregulation of mtDNMT1, although neither 12S rRNA nor 16S rRNA display significant differences. Interestingly, a stretch of mtDNA within the 16S rRNA gene devoid of CpGs (“No CpG”) displayed significant increases in methylated cytosine residues that appear to be catalyzed by increased mtDNMT1 (Figures 3A,B). This can be attributed to non-CpG methylation, which is known to be prevalent in mtDNA (Bellizzi et al., 2013; Patil et al., 2019; Van der Wijst et al., 2017). Notably, the most significant change in the level of methylation seemed to occur in a site-specific manner, localized at/near critical control regions within mtDNA, including the HSP and LSP as well as the origin of replication for the heavy strand (OriH), which is encompassed within our LSP amplicon (Figures 3A,B).

We asked whether this increase in 5mC surrounding the promoter regions might impact mitochondrial transcription. We employed a unique strand-specific priming strategy to generate polycistronic mitochondrial cDNA, designed to more accurately mimic the natural generation of nascent mitochondrial transcripts. Using both sense and antisense primers overlapping a region within the ATP6 gene, we synthesized cDNA from the heavy and light strands separately. As a loading and nuclear gene control, cDNA was also synthesized by random hexamers in parallel. We observed a 2-3-fold increase in transcription of heavy strand-encoded NADH dehydrogenase 1 (*MT-ND1, ND1*) and cytochrome c oxidase subunit 1 (*MT-CO1, Cox1*), which are located just downstream of the transcription termination (mTERF) site (Figure 3D). No significant change in the abundance of *12S rRNA* was detected, while *16S rRNA* transcript levels decreased slightly (Figure 3C). The abundance of light strand transcripts showed similar discordance, with increased expression of the light strand complementary to *ND4* and *ND5* and decreased *ND6* levels upon upregulation of mtDNMT1, suggesting that mtDNA methylation regulates mitochondrial transcription in a gene-specific fashion. These findings both support and expand upon our previous data (Shock et al., 2011), with a focus on the mitochondrial promoter and control regions (HSP, LSP, mTERF) and further implicate mtDNA methylation as a mechanism for regulating OXPHOS. However, because p53 also has been reported to localize to mitochondria and affect mitochondrial output (Marchenko et al., 2000), we sought an orthogonal approach to eliminate the potential confounding effects of p53 loss on mitochondrial transcription, and more clearly define the impacts of mtDNA methylation on mitochondrial function.

### 2.4 Genetic deletion of mtDNMT1 and/or DNMT3b activity decreases cytosine methylation across the D-loop control region of mtDNA

**FIGURE 4:**
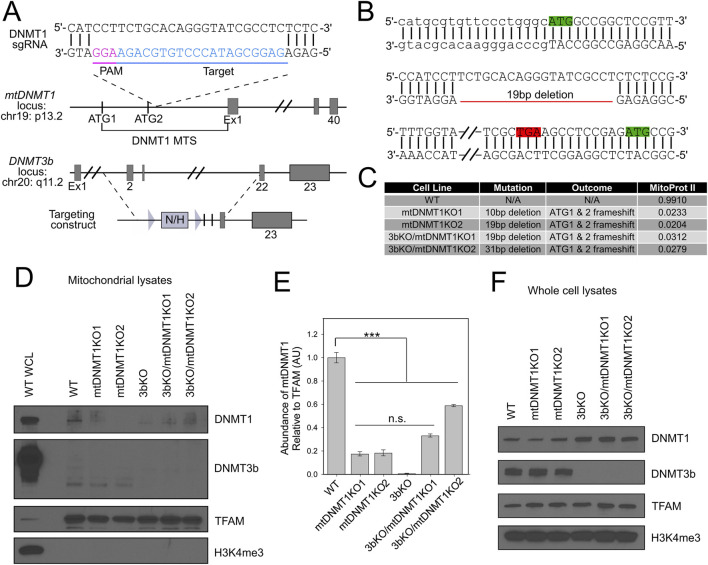
CRISPR/Cas9 targeting of the DNMT1 MTS in HCT116 WT and 3bKO cells. **(A)** Schematic of the CRISPR/Cas9-targeted mtDNMT1 locus. Bottom panel displays genetic deletion of Exons 2–21 of DNMT3b in the 3bKO parental cell line. Figure adapted from Rhee, *et al.* (Rhee et al., 2002). **(B)** Representative sequence trace for the mt1KO2 clone showing the 19bp deletion within the MTS for mtDNMT1 creates a new stop codon (TGA, red box) between the mitochondrial start (ATG, green box, line 1) and nuclear start (ATG, green box, line 2) codons. Lower case font on line 1 represents the 5′ UTR. **(C)** Table lists specific frameshift mutations and outcomes for all HCT-GE clones, including the probability of mitochondrial localization of genome-edited MTSs as predicted by MitoProtII algorithms. **(D)** Immunoblots of purified mitochondrial lysates display a substantial reduction in mitochondrial localization of DNMT1 in HCT-GE cells and complete genetic deletion of DNMT3b in the 3bKO-derived cells. WCL WT, Whole cell lysate from WT HCT116s (positive control for all antibodies); TFAM, transcription factor A of mitochondria (mitochondrial loading control); H3K4me^3^, histone 3 lysine 4 tri-methylation (nuclear contamination control). **(E)** Densitometric quantitation of DNMT1 expression in mitochondrial lysates relative to TFAM demonstrates substantial reduction in mitochondrial localization of mtDNMT1 in the HCT-GE cells. **(F)** Immunoblots of whole cell demonstrate that total DNMT protein levels remain unchanged upon CRISPR/Cas9-mediated deletion of the mtDNMT1 MTS. A DNMT3b antibody confirms the genotype of 3bKO-derived cells. TFAM, transcription factor A of mitochondria (mitochondrial loading control); H3K4me^3^, histone 3 lysine 4 tri-methylation (nuclear loading control). Error bars represent the standard deviation about the mean. Statistical significance was determined by one-way ANOVA with Tukey’s post-hoc test, ***p < 0.005.

We employed CRISPR/Cas9 genome editing to disrupt the mitochondrial targeting sequence (MTS) of mtDNMT1 in HCT116 WT and 3bKO parental cell lines (Shock et al., 2011; Mali et al., 2013) (hereafter collectively referred to as HCT-GE cells). The single guide RNA (gRNA) was strategically placed between the mitochondrial and nuclear DNMT1 translation start sites (Figures 4A,B, green boxes) with the goal of directing Cas9 cleavage within the MTS and disrupting the mtDNMT1 isoform without impacting expression of nuclear DNMT1. The gRNA was synthesized by extension of overlapping oligonucleotides, ligated into the gRNA expression vector (Mali et al., 2013) and all final constructs were verified by sequencing. WT and 3bKO HCT116 cells were transfected with gRNA and Cas9 expression plasmids and selected with puromycin. We employed the SURVEYOR nuclease assay to detect indel-mutated clones, which were extensively sequenced to reveal the specific genotype (Figures 4B,C). Clones were selected that carried 10-31bp deletions within the mtDNMT1 MTS, which created a frameshift mutation, abolishing the mitochondrial ATG (Figure 4B, green box, line 1) and generating a new stop codon upstream of the transcription start site for nuclear DNMT1 (Figure 4B, red and green boxes, line 3). MitoProtII algorithms predicted the edited MTSs would localize to mitochondria with only 2%–3% probability, as opposed to a 99% probability of mitochondrial translocation for the WT mtDNMT1 sequence (Figure 4C). The HCT-GE clones proliferated normally without extensive cell death, and displayed morphology reminiscent of their WT and 3bKO progenitor lines.

Immunoblot analysis of subcellular fractions from parental and genome-edited cell lines confirmed a reduction in mitochondrial DNMT1 expression in the mt1KO and 3bKO/mt1KO cell lines. We observed slight residual mtDNMT1 expression in mitochondrial lysates from the HCT-GE cells (Figures 4D,E, lanes 4–8), suggesting that in addition to the MTS directing its mitochondrial translocation, DNMT1 may also be chaperoned into the matrix. As expected, DNMT3b was absent in whole cell lysates from 3bKO and 3bKO/mt1KO cell lines (Figure 4F), confirming its genetic deletion. In support of our previous observations (Shock et al., 2011), the DNMT3b antibody used in this study failed to detect full-length DNMT3b in mitochondrial extracts from any of these cell lines; instead, we detect a 40 kDa immunoreactive band that we believe to be a proteolytic fragment of DNMT3b, processed upon entry into mitochondria, although its exact identity has not been confirmed (Figure 4D). Interestingly, total DNMT1 levels increased in the 3bKO cell lines, supporting a coordinated regulation of these enzymes similar to previous reports (Figure 4F). TFAM expression remains unchanged across all HCT-GE cell lines, and was used here as a mitochondrial loading control. A modified histone antibody (H3K4me3) was used as a control for nuclear contamination, and is only detected in WT whole cell lysate (Figure 4D, lane 1), demonstrating the purity of our mitochondrial lysate preparations.

**FIGURE 5:**
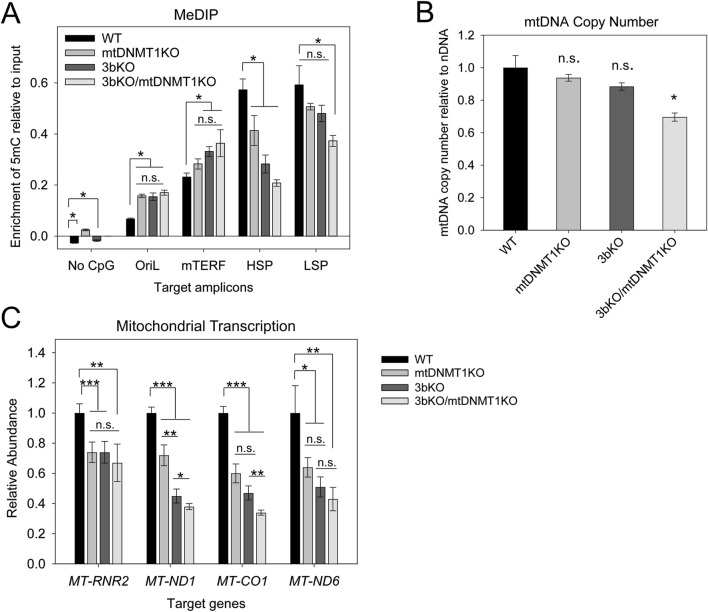
Functional consequences of genetic deletion of mtDNMT1 and/or DNMT3b in the HCT-GE cells. **(A)** MeDIP analysis shows significant alterations in 5mC enrichment across various regions of mtDNA. The most significant changes occurred within the critical control regions HSP and LSP, which displayed stepwise decreases in 5mC levels upon sequential disruption of mitochondrial DNMT activity. **(B)** ddPCR analysis detects a modest but significant reduction in mtDNA copy number upon genetic loss of both mtDNMT1 and DNMT3b. **(C)** Mitochondrial transcription originating from both HSP and LSP is dramatically reduced upon loss of mitochondrial methylation. OriL, origin of light strand replication; mTERF, termination factor site; HSP, heavy strand promoter; LSP, light strand promoter; ND1, mitochondrial NADH dehydrogenase 1; Cox1, mitochondrial cytochrome oxidase 1; ND6, mitochondrial NADH dehydrogenase 6. Experiments consisted of at least two biological replicates, each with at least three technical replicates. Error bars represent the standard deviation about the mean. Statistical significance was determined by Welch’s two-sample, two-tailed t-test, *p < 0.05, **p < 0.005, ***p < 0.0005, n. s., not significant.

MeDIP analyses confirmed a stepwise decrease in 5mC levels across both the LSP and, more strikingly across the HSP, upon sequential deletion of mtDNMT1 from WT and 3bKO parental cells (Figure 5A). A region of mtDNA devoid of CpGs (“No CpG”) was used as a non-methylated control and produced vanishingly low signal suggesting no 5mC modification within this amplicon. Surprisingly, methylation across the mTERF binding site increased upon deletion of the mitochondrial DNMTs, although these differences fail to reach statistical significance (Figure 5A). The origin of light strand replication (OriL) is relatively CpG-rich compared to the rest of mtDNA (carrying 8 CpG dinucleotides within a stretch of just 227bp), so we expected the overall enrichment of methylated cytosines within this region to be higher compared to other areas of the mtDNA genome when read off the same qPCR standard curve. However, the relative abundance of 5mC in WT HCT116 cells was on the order of 5-fold lower than across the HSP and LSP promoter regions, suggesting that low levels of methylation at the OriL may be required for light strand replication.

Additionally, MeDIP analyses of murine ES cells lacking one or both alleles of DNMT1 (DNMT1^+/−^ and DNMT1^−/−^, respectively) display a significant reduction in the level of 5mC across 12S and 16S rRNA regions (Supplementary Figure S4A). Taken together, these data confirmed that genetic deletion of DNMT1 and/or DNMT3b produced measurable differences in cytosine methylation across critical control regions of mtDNA and prompted our use of these cell lines for mitochondrial function studies.

### 2.5 Loss of methylation results in modest reductions in mtDNA content and dramatic reductions in mitochondrial transcription

To precisely quantify any potential effects of mitochondrial methylation on mtDNA copy number, a highly sensitive droplet digital PCR (ddPCR) method was employed (Hindson et al., 2013; Memon et al., 2017). Total genomic DNA isolated from the HCT-GE cells was used as the DNA template in ddPCR to determine the absolute quantification of mtDNA (measured using MT-CO1/Cox1 primers) relative to a nuclear DNA loading control (β-globin). We observed a small stepwise decrease in mtDNA copy number upon each compounding genetic insult, with the 3bKO/mt1KO cells exhibiting a significant 25% reduction compared to the WT cells (Figure 5B). Compared head-to-head with traditional qPCR (Supplementary Figure S5A), we found ddPCR to be optimal for determining mtDNA copy number in our cell lines, with greater precision and less variation between technical replicates. Both methods demonstrated the same stepwise trend, with modest reductions in mtDNA content upon genetic loss of mtDNMT1 and/or DNMT3b, confirming a role for cytosine methylation in regulating mtDNA replication.

The same priming strategy as described above (Figure 3) was used to synthesize polycistronic mitochondrial cDNA to probe the consequences of mitochondrial DNMT loss on transcription. Mitochondrial transcription was dramatically affected upon deletion of mtDNMT1 and/or DNMT3b. In cells lacking mtDNMT1, we saw a 25%–40% decrease in transcription of mtDNA-encoded OXPHOS genes, including *ND1* and *Cox1* on the heavy strand, as well as *ND6* on the light strand (Figure 5C). Genetic deletion of DNMT3b resulted in a striking 50%–60% reduction in transcription of these genes, and the compounded loss of mtDNMT1 (in the 3bKO/mt1KO cells) even further reduced mitochondrial transcription to only 35%–40% of WT levels (Figure 5C). Moreover, the differences in mitochondrial transcription were still statistically significant (∼50% reduction from WT), even after accounting for the observed differences in mtDNA content (Supplementary Figure S5B). Thus, the methylation-mediated effects on mitochondrial transcription are not solely due to a reduction in DNA template, suggesting the impacts on transcription and replication mechanisms are independent.

## 3 Discussion

Our laboratory was the first to identify a mitochondrial isoform of the most abundant nuclear DNA methyltransferase enzyme (mtDNMT1) that translocates to the mitochondrial matrix and binds to mtDNA in regions critical for mitochondrial transcription and replication (Shock et al., 2011). However, the critical question of whether mtDNMT1 retained enzymatic activity and was capable of catalyzing cytosine methylation within mitochondria was unknown. Likewise, the presence of *de novo* DNA methyltransferase, as well as TET demethylase, enzymes in mitochondria have long been debated, as neither DNMT3a, DNMT3b, nor the TETs contain a recognizable MTS, in contrast to mtDNMT1. The current work addressed these questions with rigorous genetic and innovative technical approaches, and showed that mtDNMT1 is catalytically active (Figures 1D–F) and appears to partner with DNMT3b to methylate mtDNA (Figure 1G). This cooperative relationship was first observed using mitochondrial extracts from murine embryonic stem (ES) cells, carrying catalytic deletions of each of the three catalytically active DNMTs individually, and all three in combination (Figure 1G; Okano et al., 1999; Tsumura et al., 2006): enzyme assays demonstrated clear contributions to the total mitochondrial methylation activity by both DNMT1 and DNMT3b, but not DNMT3a. We confirmed that DNMT3b makes a substantial contribution to mtDNA methylation in our family of genome-edited HCT116 cells, where loss of exons 2–21 of DNMT3b resulted in reduced mitochondrial methylase activity (Figure 1G), a coordinated drop in mtDNA 5mC levels (Figure 5A) and staggering decreases in mitochondrial transcription (Figure 5C), all supporting a role for DNMT3b in active methylation of mtDNA and regulation of mitochondrial transcription. The additional deletion of mtDNMT1 in 3bKO HCT116 cells even further exacerbated the effects of mtDNA hypomethylation, defined by significant reductions in mtDNA copy number and mitochondrial transcription (Figures 5B,C). These findings, in both normal and cancer cell contexts, and across both human and mouse species, suggest that a functional cooperation exists between mtDNMT1 and DNMT3b in the mitochondria, perhaps akin to their tasks in the nucleus.

Several attempts have been made to pinpoint the mechanism(s) responsible for generating 5hmC in mtDNA, and efforts have focused on the TET family of dioxygenase enzymes: TET1, TET2 and TET3. Both Dzitoyeva et al. (2012) and Bellizzi et al. (2013) reported mitochondrial localization of TET1 and TET2 by immunoblotting and immunofluorescence assays and found an association between increased mitochondrial TET2 and 5hmC levels in the aging brain. Likewise, Ji, et al., also determined that increased mitochondrial TET2 expression was responsible for increased 5hmC in mtDNA, and this was associated with increased mitochondrial mRNA and changes in cellular ATP levels (Ji et al., 2018). While these findings were compelling, it remained unclear which, if either, enzyme was active in mitochondria. In the present work, we directly measured TET-like hydroxymethylase activity originating from within highly-purified mitochondria (Figures 2A,B), demonstrating that the enzymatic machinery for active DNA demethylation is both present and functional in the mitochondrial matrix. Despite identifying a putative MTS just upstream of the coding sequence for TET1 with a high probability of mitochondrial translocation, we did not observe that TET1 gains access to the mitochondrial compartment (Figure 2C). However, our immunoblots detected mitochondrial localization of TET2 in HeLa cells that was resistant to trypsin digestion, suggesting that it is protected within the organelle (Figure 2D). This finding leads us to predict that mitochondrial 5hmC arises either by TET2 oxidation of 5mC or through another as yet unidentified mechanism. Further experimentation is needed to explore the mechanism of mitochondrial TET2-catalyzed generation of 5hmC in mtDNA.

A handful of mechanistic studies have examined the functional impacts of altering mtDNA methylation (Shock et al., 2011; Saini et al., 2017; Dostal and Churchill, 2019; Maresca et al., 2020; Patil et al., 2019; Van der Wijst et al., 2017); some conclude that increasing mitochondrial 5mC levels, particularly within the displacement (D-) loop control region, negatively impacts transcription (Saini et al., 2017; Patil et al., 2019; Van der Wijst et al., 2017). Such findings are consistent with the paradigm of epigenetic regulation in the nucleus, where increased 5mC at or near CpG-island promoters serves to silence transcription of the associated gene. However, mtDNA has features that are vastly different from the linear chromosomes found in the nucleus. In addition to lacking histones, the sequence of mtDNA does not contain CpG-island promoters; rather, both the HSP and LSP exhibit roughly the same CpG density as the rest of the mitochondrial genome (∼2.6–4.3%). Overall, mtDNA exhibits CpG suppression, and is rather CpG-sparse, with only 433 CpGs in the entire 16.5 kb human mitochondrial genome. These unique characteristics prompted us to ask whether epigenetic modification of mtDNA might have distinct functional consequences. Quite strikingly, our data suggest a novel mode of epigenetic regulation in mitochondria, whereby increased 5mC in/around mitochondrial promoters plays an activating role in transcription. *In vitro* assays recently published by Dostal, et al. suggest that hypermethylation of mtDNA promoter regions increased mitochondrial transcription by way of strengthening specific TFAM binding at transcription initiation sites (Dostal and Churchill, 2019). Our *in vivo* data using extracts from live cells supports such a mechanism whereby increasing 5mC at the HSP enhanced transcription (Figure 3D) and concordantly, decreasing 5mC across the HSP and LSP dramatically reduced transcription of both strands (Figure 5C). Considering that even slight deviations in TFAM levels can impact transcriptional activity, we verified that global TFAM expression remains unchanged in our HCT-GE cells (Figures 4D–F). However, more work is needed to determine whether the significant transcriptional defects displayed by our HCT116 genome-edited cells can be explained by differences in TFAM localization, specific binding to promoters and influences on overall mtDNA topology. We predict that mitochondrial 5mC and 5hmC modifications exert their effects by influencing mtDNA structure and transactions with the transcription and/or replication machineries, and this provides an interesting area for future research.

Patil, et al. recently described that knockdown of total DNMT1 and DNMT3b in MCF10A breast epithelial cells resulted in reduced global mtDNA methylation that was associated with increased mRNA expression of several mitochondrial genes across both heavy and light strands (Patil et al., 2019). This is in contrast to our findings, yet it provides the opportunity to highlight a crucial difference in our approach to quantifying mitochondrial gene expression. Here, we describe a novel strand-specific priming strategy to generate either light- or heavy-strand mitochondrial cDNA individually. Unlike the traditional approach of quantitating random hexamer-primed cDNA, which takes an average of the transcription levels across both strands, our strand-specific priming method more accurately mimics the bidirectional synthesis of nascent polycistronic mitochondrial RNA and has the potential to reveal greater underlying differences in mitochondrial transcription. Additionally, our total RNA is carefully pre-digested with DNase as a quality-control step to ensure the complete removal of contaminating mtDNA (which almost identically resembles mtRNA, with no introns or non-coding regions) prior to reverse transcription and cDNA synthesis. These key differences in methodology provide a definitive view of mitochondrial gene expression following genetic manipulation of mtDNMT levels.

DNMT1 is essential for cell survival, and while this feature makes it an extremely important target of interest, it has also complicated research investigating its function (Egger et al., 2006; Chen et al., 2007; Swiech et al., 2015; Liao et al., 2015; Smits et al., 2019). Although various cell lines carrying genetic alterations in DNMT1 exist, they are either hypomorphic (Egger et al., 2006), or contain deletions in the catalytic domain, which also affects nuclear epigenetic modifications (Okano et al., 1999; Rhee et al., 2002; Egger et al., 2006; Chen et al., 2007). Several groups have attempted to target the DNMT1 locus by CRISPR/Cas9-mediated genome editing (Swiech et al., 2015; Liao et al., 2015; Smits et al., 2019) and were met with only limited success. For instance, Smits, et al. reported residual DNMT1 transcript and protein expression arising from truncated proteins and intron retention with one of their frameshift DNMT1 KO mutant lines, which also exhibited partial preservation of methyltransferase activity (Smits et al., 2019). We generated a set of cell lines specifically deleting the MTS of DNMT1, with the goal of abolishing only mitochondrial DNMT1 activity, without impacting the nuclear isoform. We appear to have significantly reduced, but not completely eliminated, mtDNMT1KO expression and enzymatic activity in our CRISPR-targeted mtDNMT1KO subclones (Figures 4, 5), suggesting that DNMT1 activity in mitochondria may also be essential for cell survival. Remarkably, even a modest reduction in mtDNMT1 results in severe disruptions to mitochondrial function: 5mC levels, mtDNA transcription, and replication (Figure 5).

Further supporting an essential role for DNMT1 in mitochondria is the residual presence of DNMT1 in purified mitochondrial extracts from our HCT-GE cells. This raises the possibility that DNMT1 is chaperoned into mitochondria, either with or without a functioning MTS (Figure 4D). Several members of the heat shock protein family have been identified as molecular chaperones, involved in stabilizing nuclear proteins destined for mitochondria and assisting with protein re-folding after their import (Shin et al., 2021). Likewise, mitochondrial outer membrane proteins, like TOMM70, act as mitochondrial surface receptors by recruiting and tethering cytosolic chaperones to the organelle for efficient protein import (Backes et al., 2021). Another unconventional mode for targeting proteins to mitochondria involves physical contact sites with membranes from other organelles such as the ER, often termed mitochondria-associated membranes (MAMs). Close cooperation between neighboring organelles thus facilitates the exchange of proteins and metabolites (Fan and Simmen, 2019). Whether mtDNMT1 might utilize an alternative mechanism for mitochondrial import remains an open question for further investigation.

In addition to its well-characterized role as the maintenance DNA methyltransferase, DNMT1 also has been observed to bind specific mRNA transcripts and enhance stability of the associated genes through m^5^C RNA methylation (Wang et al., 2025; Di et al., 2013). Interestingly, many of the DNMT1-bound mRNAs were implicated in metabolic regulation and mitochondrial homeostasis, which constitutes an additional link between DNMT1 and mitochondrial function (Wang et al., 2025). While the focus of the current study was on DNMT1-mediated mtDNA methylation, the possibility of DNMT1 impacting mitochondrial function through RNA-level epigenetic modifications will be an attractive topic for future investigation.

Although the vast majority of mammalian cytosine methylation occurs in a CpG dinucleotide context, several reports have identified an appreciable level of non-CpG methylation, occurring at CpA, CpC or CpT sites that accumulate in the developing brain (Dzitoyeva et al., 2012; Lister et al., 2013; Kinde et al., 2015; Arand et al., 2012). Recent evidence has emerged that mtDNA also contains non-CpG methylation that may exceed the levels of CpG methylation (Bellizzi et al., 2013; Patil et al., 2019; Van der Wijst et al., 2017). We considered the implications of non-CpG methylation in light of our data. While the antibodies used in our MeDIP assays are specific for either 5mC or 5hmC modifications, with no measurable cross-reactivity, they do not distinguish between 5mC occurring in different dinucleotide contexts. Therefore, it is quite possible that a proportion of the cytosine methylation we detect in our MeDIP analyses can be ascribed to non-CpG methylation. This likely explains the notable difference in the levels of 5mC and 5hmC measured across the “No CpG” amplicon in HCT116 cells when mtDNMT1 is upregulated (p53^−/−^) relative to control (p53^+/+^) (Figures 3A,B). The *de novo* DNMTs (DNMT3a and 3b) have been shown to catalyze cytosine methylation in a non-CpG context (Arand et al., 2012), suggesting 5mC modifications in mtDNA could arise through DNMT3b activity in mitochondria. Regardless of dinucleotide context, our data clearly demonstrates that the total mitochondrial methylation profile is altered upon genetic disruption of the mtDNMTs (Figures 3–5), and these epigenetic changes impact processes central to mitochondrial function.

With mitochondrial transcription functioning at only 25%–60% of WT levels, we expect the mt1KO and 3bKO/mt1KO cells to exhibit severe metabolic deficiencies, resulting from failure to synthesize essential mtDNA-encoded subunits of the respiratory chain complexes. We saw significant decreases in mitochondrial gene expression of *ND1* and *ND6*, whose proteins form subunits of Complex I, as well as *Cox I*, a subunit of Complex IV of the electron transport chain (ETC) (Figure 5C). Stoichiometric imbalances in mtDNA-encoded ETC, components have been shown to cause impaired mitochondrial respiration and metabolic remodeling, which induces oxidative stress and leads to progressive accumulation of mtDNA damage (Nikkhanen et al., 2016; Masand et al., 2018). Importantly, downregulation of mitochondrial genes has been associated with invasive and metastatic signatures and worse clinical outcomes in numerous different cancer types (Gaude and Frezza, 2016). Thus, further examination of the functional consequences of altering mtDNA methylation, with a particular focus on the impacts to cellular metabolism, is warranted.

Here, we report that the enzymatic machinery capable of both writing and erasing cytosine methylation is present and active in mitochondria. 5mC modifications are catalyzed through the cooperation of two distinct DNMT enzymes: DNMT1 and DNMT3b. While we cannot rule-out the presence of other 5mC-oxidation mechanisms, our data suggest that 5hmC modifications arise through the hydroxymethylase activity of TET2. Genetic deletion of the mitochondrial DNMTs results in an altered mtDNA methylation profile, particularly across the critical control regions housing the transcriptional promoters HSP and LSP. This reduction in mtDNA 5mC levels leads to dramatic reductions in mitochondrial transcription and mtDNA replication, which appear to occur independently of one another. Our data support a role for epigenetic regulation of mitochondrial physiology as a mechanism for maintaining cellular health and homeostasis, and implicate aberrant mtDNA methylation in disease.

## 4 Experimental procedures

### 4.1 Materials

HCT116 p53^+/+^ and HCT116 p53^−/−^ and HCT116 3bKO cells were obtained from Dr. Bert Vogelstein, Johns Hopkins University (Baltimore, MD). Primary MEFs (p53^+/+^ and p53^−/−^) were prepared from E12.5-E13.5 mouse embryos. WT HCT116 cells and HEK293 cells were obtained from American Type Culture Collection (ATCC, Manassas, VA). Murine embryonic stem cells were obtained from Dr. En Li, Harvard University (Boston, MA). The hCas9 and gRNA plasmids were generated in the Church laboratory (Kinde et al., 2015) and purchased from Addgene (#41815 and #41824).

Protein G and IgG sepharose beads were purchased from GE Healthcare (Uppsala, Sweden). AcTEV Protease was purchased from Invitrogen (ThermoFisher). DNA oligo primers (25nmoles) were designed using OligoAnalyzer software from Integrated DNA Technologies (IDT, Coralville, IA) and purchased from Eurofins Genomics (Louisville, KY), unless described otherwise. SsoAdvanced Universal SYBR Green Supermix was purchased from Bio-Rad and used for all qPCR and RTqPCR analyses. A Bioruptor water bath sonicator (Diagenode, Denville, NJ) was used to shear gDNA to a range of 400-700bp fragments. BioRad DNA Engine Peltier thermal cyclers, fitted with Chromo 4 Real-Time Fluorescence Detector attachments were used for all studies involving qPCR. Real-time qPCR data was analyzed with Opticon Monitor 3 Software. Beckman centrifuges (Palo Alto, CA) were used for all differential centrifugation studies: Beckman J6-MI, fitted with JS 4.2 rotor, and a Beckman J2-HC, fitted with JA-17 rotor.

DNMT1 antibody was purchased from Abcam (Cambridge, MA). DNMT3b antibody was purchased from Cell Signaling. TAP-tag antibody was purchased from Open Biosystems (#CAB1001). 5-methylcytosine (#61255) and 5-hydroxymethylcytosine (#39769) antibodies were from Active Motif (Carlsbad, CA). All other reagents of the highest available grade were from Fisher Scientific (Pittsburgh, PA) or Sigma-Aldrich (St. Louis, MO).

### 4.2 Cell culture

WT and p53^−/−^ MEFs were grown at 37 °C in 10% CO_2_, in DMEM medium (Gibco/Invitrogen) supplemented with 10% FBS. All HCT116 cells were grown at 37 °C in 5% CO_2_, in RPMI 1640 medium supplemented with 10% FBS. Murine ES cells were grown in a feeder monolayer of MEFs at 37 °C in 10% CO_2_, in DMEM medium, supplemented with 10% FBS, 25 mM HEPES, 5.6 mM D-glucose, 4.0 mM glutamine, 1.0 mM sodium pyruvate, 0.1 mM non-essential amino acids, 0.1 mM β-mercaptoethanol, and 1 μg/mL leukocyte inhibitory factor (LIF, Gemini). All cell media and supplements were obtained from Gibco/Invitrogen, unless otherwise noted, and FBS was obtained from Gemini.

### 4.3 Generation of the HCT116 DNMT1-TAP cell line

The HCT116 DNMT1-TAP cell line was generated in the WT HCT116 background by targeting Exons 39–40 of the DNMT1 C-terminal endogenous locus through homologous recombination as previously described (Maresca et al., 2020). Left and right homology arms (LHA and RHA, respectively) were amplified from the genomic locus of DNMT1 using total gDNA from HCT116 cells as a template. The TAP-tag was amplified from the pZome-1C plasmid; the Neo^R^ selection cassette was excised from the pNeDaKO-Neo vector by restriction digest with the *Pvu*I endonuclease. The TAP-tag targeting construct was generated by joining the LHA and RHA with the TAP-tag and Neo^R^ cassette by a series of fusion PCRs. The final targeting construct was cloned into the pAAV-MCS vector and co-transfected into HEK293 cells, along with pAAV-RC and pHelper plasmids (Stratagene AAV Helper-Free System) for packaging of recombinant adeno-associated virus. Viral supernatant from HEK293 cells was used to infect two T-75 flasks of sub-confluent WT HCT116 cells for 48hrs. Transduced cells were plated by limiting dilution and selected for Neo^R^. Positively-recombined Neo^R^ clones were screened by PCR and sequenced to ensure proper integration of the TAP-tag. Selected clones were transfected with Cre recombinase to excise the *loxP*-floxed Neo^R^ cassette. Final clones were again screened by PCR and genotyped by Southern blot.

### 4.4 CRISPR/Cas9 genome editing of the mtDNMT1 MTS

The mtDNMT1KO and mtDNMT1KO/3bKO cells were generated in WT and 3bKO HCT116 parental cells, obtained from Bert Vogelstein (Johns Hopkins University, Baltimore, MD) (Dzitoyeva et al., 2012). The MTS of mtDNMT1 was disrupted by CRISPR/Cas9 genome editing, as described (Di et al., 2013). Parental WT and 3bKO HCT116 cells were transfected with the sgRNA shown in Supplementary Figure S2, and the hCas9 plasmid obtained from the Church lab (Mali et al., 2013). Targeted cells were plated by limiting dilution and screened for Cas9 activity by the T7 SURVEYOR Assay. The genotype of selected clones was verified by sequencing. A 19bp deletion within the MTS removed a large portion of the mtDNMT1 pre-sequence and also resulted in a frameshift, introducing a Stop codon just upstream of ATG3, the translational start site for nuclear DNMT1 (Supplementary Figure S2). Two independent clones for each modification were used as internal controls in all experiments.

### 4.5 Mitochondrial isolation

Cells were harvested from sub-confluent dishes by scraping on ice and pelleting by centrifugation. The whole cell pellet from approximately 50–60 million cells was resuspended in 3 mL of mitochondrial homogenization buffer (0.25M sucrose, 10 mM Tris-HCl, pH 7.0, 1 mM EDTA, pH 6.8) containing Complete EDTA-free Protease Inhibitor Cocktail tablets (Roche). An aliquot (5%–10%) of this resuspended cell volume was retained in a separate tube to generate the whole cell lysate. Cells were lysed in SDS Lysis buffer (62.5 mM Tris pH 6.8, 5% glycerol, 2% SDS, 5% β-mercaptoethanol, and 1x complete protease inhibitor cocktail) at a volume 7.5x the pellet weight. The remainder of the resuspended cell volume (90%–95%) was transferred to a pre-chilled 7 mL glass dounce homogenizer (Wheaton) and subjected to three rounds of dounce homogenization, followed by differential centrifugation to separate the nuclear fraction from the post-nuclear fraction (containing mitochondrial and cytosolic fractions). The post-nuclear supernatant was centrifuged at 10,000xg for 15 min at 4 °C to pellet the mitochondria. Crude intact mitochondrial pellets were washed twice with 1 mL of mitochondrial homogenization buffer in the presence of complete protease inhibitors, and pelleted by centrifugation at 10,000xg for 10 min at 4 °C after each wash. The mitochondrial pellets were washed once with 1 mL of mitochondrial homogenization buffer in the absence of protease inhibitors, and resuspended in Trypsin Digestion buffer (10 mM HEPES-KOH, pH 7.4, 250 mM sucrose, 0.5 mM EGTA, 2 mM EDTA, 1 mM DTT) in a volume 20x the pellet weight. Trypsin-EDTA (Gibco) was added to achieve a final concentration of 10 μg/mL, and samples were incubated at room temperature for 20 min with occasional inversion to mix. Bovine trypsin inhibitor (Sigma) was added to a final concentration of 10 μg/mL, tubes were inverted several times to mix, and incubated on ice for approximately 10 min. Intact mitochondria were pelleted by centrifugation at 10,000xg for 10 min at 4 °C, and washed twice more with 1 mL of mitochondrial homogenization buffer, this time in the presence of both complete protease inhibitors, and 10 μg/mL bovine trypsin inhibitor. Trypsin-treated mitochondrial pellets were weighed and lysed in a volume of SDS Lysis Buffer 10x the pellet weight. The cytosolic fraction was obtained by adding an equal volume of ice-cold 20% TCA to the post-mitochondrial supernatant. The sample was mixed well, and incubated on ice for 30 min. Cytosolic proteins were pelleted at 6,000xg for 15 min at 4 °C, washed 2–3 times with 3–5 mL of ice-cold acetone, each time spinning at 6,000xg for 5 min at 4 °C to collect the pellet. After the last wash, the cytosolic pellet was allowed to air-dry on ice for approximately 5 min, then dissolved in 0.5 mL SDS lysis buffer.

### 4.6 Mitochondrial DNA nucleoid extraction

Mitochondrial DNA nucleoids were isolated from purified mitochondria using an adaptation of the protocol described by Garrido et al. (2003) . The intact trypsin-treated pellet was resuspended in 1.5x NE2 buffer (0.25M sucrose, 20 mM Tric-HCl, pH 7.5, 2 mM EDTA, 7 mM β-mercaptoethanol and complete protease inhibitors), and diluted with an equal volume of 0.5X NE2 buffer to a final concentration of 5–7 mg/mL of mitochondrial protein. Mitochondria were lysed by adding 20% NP-40 to a final concentration of 0.5%. 1M Spermidine (Sigma) was added to a final concentration of 3 mM to bind and precipitate the mtDNA. Mitochondrial extracts were incubated on ice for 15 min to ensure complete lysis, then split evenly between two 1.5 mL microcentrifuge tubes: one for immunoblot analysis, the other for enzyme assay. The mitochondrial lysates were sub-fractionated into soluble (“S”) and insoluble pellet (“P”) fractions by centrifugation at 12,000xg for 20 min at 4degC.

### 4.7 Purification of mtDNMT1-TAP enzyme

Roughly 1 × 10^7^ HCT116 DNMT1-TAP cells were harvested by scraping and pelleted by centrifugation. Trypsin-treated intact mitochondria were isolated as described above. Mitochondrial pellets were lysed with mtDNMT1 Lysis Buffer (50 mM Tris-HCl, pH 7.5, 1 mM DTT, 1 mM EDTA, 5% glycerol, 0.1% Tween 20, and 1x complete protease inhibitor cocktail) and sonicated using a Misonix Sonicator 3,000 fitted with the probe attachment, cycling between 1s on, 5s off, for a total of 25 cycles. The lysate was incubated on ice for 15 min to ensure complete lysis.

mtDNMT1-TAP was purified following the TAP-tag purification scheme described by Rigaut, et al. (Dostal and Churchill, 2019). IgG beads were washed three times with mtDNMT1 lysis buffer, each time pelleting the beads by centrifugation at 250 *g* for 2 min at 4 °C. Washed and equilibrated beads were resuspended into a 50% slurry with mtDNMT1 lysis buffer. 100µL of bead slurry was added per ∼8 mL of mitochondrial lysate, and incubated overnight at 4 °C with end-over-end rotation.

Following overnight incubation, the beads were pelleted by centrifugation at 250 *g* for 2 min at 4 °C. Approximately 1 mL of the unbound flowthrough was collected and retained for analysis. IgG beads were transferred to a 1.5 mL microcentrifuge tube and washed three times with 1 mL of mtDNMT1 Lysis buffer, each time pelleting the beads at 250xg for 2 min at 4 °C. The beads were then washed three times with 0.5 mL 1x TEV buffer, spinning at 250xg for 2 min at 4 °C in-between each wash.

Washed IgG beads were resuspended in 300 µL of 1x TEV buffer, and 100U of AcTEV Protease was added to the tube. The reaction was incubated at room temperature for 2.5hrs, rotating end-over-end. After 2.5hrs, the reaction was spiked with 50U additional AcTEV Protease enzyme. The TEV cleavage reaction was allowed to proceed overnight at 4 °C with end-over-end rotation to permit complete cleavage of bound mtDNMT1-TAP from the IgG beads.

After overnight incubation in the presence of AcTEV Protease, the IgG beads were pelleted by centrifugation at 250 *g* for 2 min at 4 °C. The supernatant, containing the cleaved mtDNMT1-TAP, was transferred to a clean 1.5 mL microcentrifuge tube. The concentrations of EDTA and NaCl were adjusted to 1 mM EDTA and 200 mM NaCl, and glycerol was added to 50% of the total volume to more closely match the storage conditions for the recombinant hDNMT1 enzyme (New England Biolabs). IgG beads containing residual (uncleaved) bound material were boiled in SDS Lysis buffer for 5 min, pelleted by centrifugation, and the supernatant was collected and saved for analysis.

### 4.8 SDS-PAGE and immunoblotting

Each subcellular fraction (whole cell lysate, cytosolic and mitochondrial) was collected in a pre-weighed 1.5 mL microcentrifuge tube, and weighed to calculate the appropriate volume of SDS Lysis Buffer (62.5 mM Tris-HCl, pH 7.5, 5% glycerol, 2% SDS, 5% β-mercaptoethanol, 1x complete protease inhibitor cocktail (Roche)) to add to each sample. SDS Lysis buffer was added at 7.5x the whole cell pellet weight, and 10x the cytosolic and mitochondrial pellet weights. Whole cell and cytosolic lysates were passed through a 21G needle fitted with 1 mL syringe approximately 20 times to shear the gDNA and create a homogeneous mixture. Lysates were aliquoted and stored at −80 °C until use.

Protein concentrations were calculated using a Bradford Protein assay against a standard curve generated by absorbance values measured for known concentrations of BSA, read at 595 nm. Protein was loaded onto SDS-PAGE gels to approximate equal cell equivalents, so that an equal signal for each compartment-specific antibody was obtained (whole cell lysate: 75 µg, cytosolic lysate: 25 µg, mitochondrial lysate: 18 µg). The appropriate amount of total protein from each cell fraction was diluted in an equal volume of Laemmli sample buffer containing 5% β-mercaptoethanol, boiled for 5 min, and quickly spun to collect the samples. Boiled lysate samples were loaded into the wells of SDS-PAGE 4%–15% gradient gels (BioRad Tris-HCl Ready gel) and resolved by electrophoresis at 150V for approximately 1 h.

Resolved proteins were transferred onto an Immobilon PVDF membrane (EMD/Millipore), soaked in 100% methanol and sandwiched between 2 sheets of pre-cut Whatman paper (3 mm Chromatography paper). Each sheet of Whatman paper was soaked in 1x Transfer buffer (25 mM Tris-HCl, pH 8.3, 192 mM glycine, 20% methanol, 0.1% SDS) and rolled smooth to remove any bubbles. The transfer sandwich was lowered into a wet transfer tank was filled with 1x Transfer buffer. The transfer apparatus was connected to a power supply and run at 100V for 50–60 min to transfer all proteins onto the PVDF membrane.

Membranes were blocked in T20 StartingBlock blocking buffer (ThermoFisher), either for 1 h at room temperature or overnight at 4 °C, with gentle rocking on a platform shaker. Blots were washed with 1x TBS-T (0.5M Tris-HCl, pH 7.5, 0.14M NaCl, 2.7 mM KCl and 0.1% Tween 20) three times for 5 min each with vigorous shaking. Primary and secondary antibodies were diluted in StartingBlock blocking buffer under the conditions optimized for each antibody. Membranes were incubated in primary and/or secondary antibody for 1 h at room temperature with gentle shaking. Following both primary and secondary antibody incubation, membranes were washed with 1x TBS-T three times for 10 min each with vigorous shaking. All secondary antibodies were conjugated with horseradish peroxidase to allow for chemiluminescent detection using the SuperSignal West Pico (Pierce) and West Dura (Pierce) Chemiluminescent Substrate kits according to manufacturer’s instructions. Blots were developed either using autoradiography film (ISC Bioexpress) and a Konica SRX-101A developer, or on the Licor Odyssey Imaging System using ImageStudio software.

### 4.9 DNA methyltransferase assays

*GlaI Cleavage Assay*. We designed and optimized a fluorescence-based enzymatic assay similar to the DNA methyltransferase assay described by Wood, et al. (Hao et al., 2020), utilizing the Chromo4 fluorescence detection system fitted on our BioRad Real-Time PCR machines. A schematic diagram of the assay is shown in Figure 1.

All enzyme assays were prepared in a total volume of 100µL, consisting of 10 mM Tris-HCl, pH 7.5, 5 mM MgCl_2_, 1 mM DTT, 5% glycerol, 0.1 mg/mL BSA, 25–100 mM NaCl and 0–1 mM S-adenosylmethionine (SAM). Two stocks of Buffer A were prepared and aliquoted for these experiments: one stock of Buffer A-25, containing 25 mM NaCl, and Buffer A-100, which contains 100 mM NaCl. Fresh stocks of 1x SEB Buffer (10 mM Tris-HCl, pH 8.5, 5 mM MgCl_2_, 10 mM NaCl, 1 mM β-mercaptoethanol), 0.8U/µL GlaI in 1x SEB Buffer, 1 mM SAM, as well as Oligo1 and Oligo2 (various concentrations) were made before each reaction was assembled.

All reactions were run in duplicate, using low-profile, white PCR strips, sealed with flat cap strips (BioRad). Reaction tubes were quickspun to collect all contents at the bottom of the tubes. A BioRad Peltier Thermal Cycler was pre-warmed to 37 °C prior to loading reactions into the machine. The length of reactions ranged from 1 to 6hrs, with fluorescence readings taken every 1 min.

Two fluorescently-labeled oligo probes (hemi-methylated Oligo1 and fully-methylated Oligo2) were designed that are identical except for a single methylation site at an internal CpG (Supplementary Table S1). Both oligos were labeled at their 5′ end with the fluorescein fluorophore 6-FAM and at the 3′ end with Black Hole Quencher 1 (BHQ1). Because the excitation/emission wavelengths for the 6-FAM fluorophore mimic that of SYBR Green dye used in qPCRs, the fluorescence could be measured on our real-time PCR machine. The fully-methylated Oligo2 was used as a positive control, to determine the optimal concentration of Oligo and GlaI enzyme needed to stay within the linear range of DNMT1 activity. Oligo2 calibration curves were prepared with 0 mM SAM, ± 1U GlaI, Buffer A-100 and a range of Oligo2 concentrations between 0 and 20 nM.

Selectivity of GlaI enzyme between Oligo1 (non-optimal) and Oligo2 (optimal) substrates was measured in duplicate 100 µL reactions, with 50µM SAM, 2.4U GlaI, Buffer A-100 and Oligo1 concentrations between 0.028 and 1 µM. An identical range of concentrations was tested for Oligo2, in duplicate 100 µL reactions containing 50µM SAM, 0.05U GlaI and Buffer A-100.

Non-specific GlaI cleavage of the hemi-methylated Oligo1 substrate was measured in duplicate 100 µL reactions, with 50µM SAM, 0.8U GlaI, ± 10 nM *M. Sss*I, Buffer A-100 and Oligo1 concentrations between 0.13 and 0.6 µM. All validation assays were incubated at 37 °C for 1 h, with fluorescence readings taken every 1 min.

*EpiQuik Fluorescence Assay*. Trypsin-treated mitochondrial extracts isolated from murine ES cells carrying catalytic deletions in each of the DNMTs individually and in combinations were assayed for total DNMT activity using the fluorometric EpiQuik DNMT Activity/Inhibition Assay Ultra kit (Epigentek, Farmingdale, NY). Assays were performed using 20 µg total mitochondrial extract according to the manufacturer’s protocol. Fluorescence was measured at room temperature over a 30 min period, with readings taken every 1 min.

### 4.10 TET enzyme assay

Each *in vitro* Tet enzyme assay is comprised of: a DNA template (the APC promoter), which contains methylated cytosines as the substrate, an Fe^2+−^containing salt, a co-factor essential for activity of Tet enzymes, and mitochondrial lysate as the source of Tet enzyme activity. 5µL of the trypsin-treated crude or Percoll-purified mitochondrial lysate, containing 50% glycerol, was incubated with 100 ng of methylated APC promoter template DNA in the presence of TET enzyme assay buffer (100 mM HEPES, pH 6.8, 150 µM Fe(NH_4_) (SO_4_), 4 mM ascorbate, 2 mM α-ketoglutarate) at 37 °C for 1 h. The final volume of the reaction was adjusted to 25 µL using HPLC water. Reactions were terminated by quenching on ice for 5 min 1 µg of purified recombinant TET1 C-terminal domain (Active Motif, cat # 31363) was used as a positive control. 5µL of the respective mitochondrial lysates were boiled for 10 min as a negative control. All reactions were performed alongside a corresponding No Substrate control to account for signal intrinsically obtained from mitochondrial lysates due to the presence of mtDNA.

### 4.11 5hmC slot blot

20 ng of the Tet enzyme assay reaction sample was added to 6X SSC, pH 7.0, to a final volume of 100uL. Samples were boiled for 10 min, then immediately quenched on ice for 10 min. An equal volume (100uL) of 20X SSC solution was added to each of the samples before loading them into the wells of the slot-blot apparatus. Samples were spotted onto a pre-cleaned and pre-equilibrated nitrocellulose membrane; the membrane was rinsed briefly and UV crosslinked. The membrane was blocked for 1 h in 5% milk, then incubated with anti-5hmC (Active Motif, cat # 39791, 1:500) in 5% milk for 1 h, and goat-anti-rabbit-HRP secondary (ThermoFisher, cat # 31460, 1:15,000) for 1 h. Chemiluminescent substrate (Pierce) was used to develop the blot.

### 4.12 Methyl-DNA immunoprecipitation

Total gDNA was purified from 8–10 × 10^6^ HCT116 cells using the DNEasy Blood and Tissue kit (Qiagen), according to the manufacturer’s recommended protocol. Purified DNA was Pheno/chloroform extracted, ethanol-precipitated, and resuspended in 500 µL of 10 mM Tris, pH 8.0. DNA was quantitated using the ND-1000 NanoDrop spectrophotometer. DNA was randomly sheared by sonication to an average size of 400-700bps using a Bioruptor water bath sonicator (Diagenode); DNA fragment size was verified by gel electrophoresis. A total of 4 µg of sheared genomic DNA was used as input into each MeDIP assay. The DNA was denatured by boiling for 10 min, and then immediately cooled on ice for 10 min. An aliquot (51 µL) of 10x IP buffer (100 mM NaPO4, pH 7.0, 1.4M NaCl, 0.5% Triton X-100) and 2 µg of each antibody for IP were added to the denatured DNA sample, and incubated at 4 °C overnight with end-over-end rotation. A total of three conditions were examined for each DNA sample: Nonspecific IgG (-) control (Millipore, cat # 12–371), 5mC antibody (Active Motif, cat # 39649), and 5hmC antibody (Active Motif, cat # 39770).

Lambda DNA (New England BioLabs, cat #N3011L) was sonicated using a Diagenode Bioruptor water bath sonicator at the highest pulse for 2 cycles of 15 min each, with 1 min on and 1 min off. After each cycle, the tubes were removed from the sonicator, tapped to mix and quickspun to collect all DNA at the bottom of the tubes.

Protein G sepharose beads (GE Healthcare, cat # 17–0,618–01) at a volume of 20 µL per sample, per condition, were pipeted using a 2–200 µL tip with the end cut off and pelleted by centrifugation at 4,500rpm for 5 min at 4 °C in a tabletop microfuge, to remove the 70% ethanol resuspension mix. The beads were washed with 400 µL of 1x PBS-0.1% BSA solution three times, incubating the beads with rotation for 5 min at 4 °C and spinning at 4,500rpm for 5 min at 4 °C. At the end of the third wash, the volume of pelleted beads was estimated, and an equal volume of 1x IP buffer was added to make a 50% bead slurry. BSA (5 µg per 30 µL of 50% slurry) and sonicated lambda DNA (5 µg per 30 µL of 50% slurry) were added to the bead slurry and incubated for 3 h at 4 °C with rotation to block the beads and prevent non-specific IgG binding. After blocking, the beads were again washed with 1x PBS-0.1% BSA as described above, and the pellet was resuspended in an equal volume of 1x IP buffer, and mixed by tapping the tube.

Approximately 20 µL of blocked, washed bead slurry was added to each tube containing a DNA-antibody complex. These samples were incubated for 2 h at 4 °C with rotation to allow DNA-antibody-Protein G bead complexes to form. Beads were collected by centrifuging at 4,500rpm for 5 min at 4 °C, and washed three times, each with 500 µL 1x IP buffer for 10 min at 4 °C with rotation, and spinning at 4,500rpm for 2 min at 4 °C. After the third wash, the beads were resuspended in 250 µL of Proteinase K digestion buffer (50 mM Tris, pH 8.0, 10 mM EDTA, 0.5% SDS), and mixed by tapping the tube. Seventy µg of Proteinase K (3.5 µL of 20 mg/mL stock, Bioline, cat # BIO-37037) was added to each sample, and incubated for 3 h at 50 °C with shaking to disrupt the antibody-Protein G bead interactions. Immunoprecipitated DNA was extracted with 250 µL of Phenol:chloroform:Isoamyl alcohol (25:24:1), and precipitated using 1/10th volume (25 µL) of 3M NaOAc, 40 µg of UltraPure Glycogen (2 µL of 20 μg/μL stock, Invitrogen, cat # 10814010), and 3 volumes (750 µL) of 100% ethanol per sample. The DNA pellet was allowed to air-dry at room temperature for approximately 5 min, resuspended in 75 μL TE buffer, pH 8.0, and stored at −20 °C.

Quantitative PCR using Quantitect SYBR Green mastermix and mitochondrial-specific primers (see Supplementary Table S1 for primer sequences) was performed to measure the level of enrichment of mtDNA sequences in immunoprecipitated material pulled down by each antibody: anti-5mC, anti-5hmC or nonspecific IgG. Absolute amounts of immunoprecipitated mtDNA were quantitated using a standard curve of purified mtDNA, and expressed relative to input.

### 4.13 Mitochondrial DNA copy number

Total gDNA was isolated from 8–10 × 10^6^ HCT116 genome-edited cells using the DNeasy DNEasy Blood and Tissue kit (Qiagen), according to the manufacturer’s recommended protocol, and including the RNase A treatment step. Droplet Digital PCR (ddPCR, BioRad) was performed according to manufacturer’s protocol, using EvaGreen Droplet Generation oil and EvaGreen ddPCR Supermix in 20 μL total volume per well. Each ddPCR reaction contained: 1x EvaGreen ddPCR Supermix, 100 nM of each primer, and either 25 ng of total gDNA template (nuclear reference gene, β-globin) or 50 pg of total gDNA template (mitochondrial gene, MT-COI). Data analysis and copy number calculations were performed using QuantaSoft software.

### 4.14 Mitochondrial transcription

Total RNA was isolated from 8–10 × 10^6^ cells using TRIzol Reagent, and reverse transcribed with the SuperScript III First-Strand Synthesis System, both according to the manufacturer’s instructions. Total cDNA was synthesized using random hexamers to obtain a complete representation of the transcribed genome. Strand-specific mitochondrial cDNA was synthesized using complementary gene-specific primers located within the *MT-ATP6* gene: ATP6sn for light strand cDNA synthesis, and ATP6asn for heavy strand cDNA synthesis (sequences can be found in Supplementary Table S1). Changes in mitochondrial gene expression were measured using primers specific to four different regions of the mitochondrial genome, 3 on the heavy strand: *MT-RNR2* (*16S rRNA*), *MT-CO1* (*COX1*) and *MT-ND1* (*ND1*), and one on the light strand: *MT-ND6* (*ND6*). Primer sequences are listed in Supplementary Table S1. Each dataset was normalized to the geometric mean of *GAPDH* and *β-Actin* expression. Two independent sets of biological samples were analyzed for each gene, with triplicate technical repeats in each sample. Standard deviations (SDs) were computed using the formula:

$$\left.SD={AVG}_{sample}/{AVG}_{input}\times \left(\sqrt{\left({\left({\text{SD}}_{\text{sample}}/{\text{AVG}}_{\text{sample}}\right)}^{2}+{\left({\text{SD}}_{input}/{AVG}_{input}\right)}^{2}\right)}\right)\right)$$

## Data Availability

The raw data supporting the conclusions of this article will be made available by the authors, without undue reservation.
