# Supplementary material for: Cytosine methylase and hydroxymethylase activity in mammalian mitochondria
Source: Front Cell Dev Biol. 2025 Oct 15;13:1677402. doi: 10.3389/fcell.2025.1677402 (PMC12568578; doi:10.3389/fcell.2025.1677402)
Supplement: Supplementary file 1 [file DataSheet1.pdf]

Cytosine methylase and hydroxymethylase activity in mammalian mitochondria

Supplemental Info

Table S1: Oligonucleotide and Primer Sequences

Table S1. Oligonucleotide and Primer Sequences

**Oligonucleotides**		
**CpG Methyltransferase assay**		
Oligo 1	6-FAM/CCTATGCG/5mC/ATCAGTTTTCTGATG/5mC/G/5mC/ATAGG/BHQ_1	
Oligo 2	6-FAM/CCTATG/5mC/G/5mC/ATCAGTTTTCTGATG/5mC/G/5mC/ATAGG/BHQ_1	
**CRISPR/Cas9**		
DNMT1-MTS sgRNA	GAGGCGATACCCTGTGCAGAAGG	
**Primers**		
**DNMT1-TAP Generation**	**Forward**	**Reverse**
LHA	ATACATACGCGGCCGCCCCACGTGTCTTTGTCTCAAGTCTTTC	CCATCTTCTCTTTTCCATGGATCCGTCCTTAGCAGCTTCCTCCTC
RHA	CGCCCTATAGTGAGTCGTATTACTTCTGCCCTCCCGTCACCCCTGTTT	ATACATACGCGGCCGCAGGAGGCAGAGGCTGCATTGAACG
TAP	GGACGGATCCATGGAAAAGAGAAGATGG	GCTCCAGCTTTTGTTCCCTTTAGCTAATTAGCGTCTACTTTCGGCGC
**MeDIP Primers**	**Forward**	**Reverse**
No CpG	CTGGTGATAGCTGGTTGTCCAAGA	CCTAGTGTCCAAAGAGCTGTTCCT
12S rRNA	AGTTCACCCTCTAAATCACCACG	TGACTTGGGTTAATCGTGTGACC
16S rRNA	ACCTTACTACCAGACAACCTTAGCC	TAGCTGTTCTTAGGTAGCTCGTCTGG
mTERF	AACTTCCTACCACTCACCCTAGCA	TGGATTCTCAGGGATGGGTTCGAT
HSP	TCCCACTCCCATACTACTAATCTCATC	AGGACCAAACCTATTTGTTTATGG
LSP	TCTGGCCACAGCACTTAAAC	TGTGTGCTGGGTAGGATGG
OriL	ACTCTGCATCAACTGAACGC	TTACCAGCTCCGAGGTG
**Mitochondrial Genes**	**Forward**	**Reverse**
MT-RNR1 (*12S rRNA*)	AGTTCACCCTCTAAATCACCACG	TGACTTGGGTTAATCGTGTGACC
MT-RNR2 (*16S rRNA*)	ACCTTACTACCAGACAACCTTAGCC	TAGCTGTTCTTAGGTAGCTCGTCTGG
MT-ND1 (*ND1*)	TCATCTCAACTTAGTATTATACCCACACC	TTAAGAAGAGGAATTGAACCTCTGACTG
MT-CO1 (*Cox1*)	ATTTAGCTGACTCGCCACACTCC	ATACAATGCCAGTCAGGCCACC
MT-CO2 (*Cox2*)	ACAGATGCAATTCCCGGACGTC	TGGGCATGAAACTGTGGTTTGCTC
MT-ATP6 (*ATP6*)	ATTCAACCAATAGCCCTTGGCCG	ACGTAGGCTTGGATTAAGGCGAC
MT-ND4 (*ND4*)	AGTTCACCCTCTAAATCACCACG	TGACTTGGGTTAATCGTGTGACC
MT-ND5 (*ND5*)	GAGACAAGTCGTAACATGGTAAG	GGGTAAGGTTTGCCGAGTTCCT
MT-ND6 (*ND6*)	AAACACTCACCAAGACCTCAACCC	ATTGATTGTTAGCGGTGTGGTCGG
**Nuclear Genes**	**Forward**	**Reverse**
B-Actin	TCATACTCCTGCTTGCTGATCC	CCACGAAACTACCTTCAACTCC
GAPDH	CCAGGTGGTCTCCTCTGACTTC	TTGGAGGCCATGTGGGCCATGA
**Mito Copy Number (gDNA)**	**Forward**	**Reverse**
B-globin	ATCTACTCCCAGGAGCAG	AACTTCATCCACGTTCACC

Figure S1: Sub-mitochondrial isolation of mtDNMT1-TAP

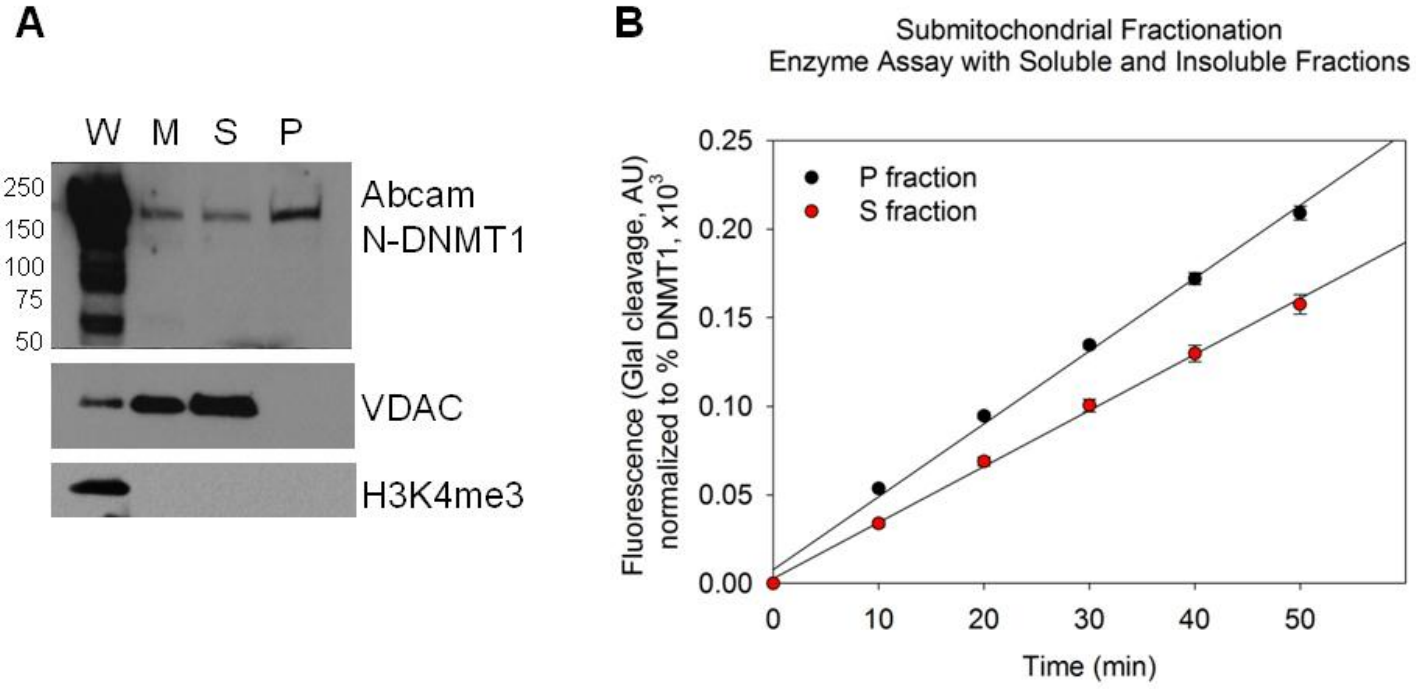

Figure S1. mtDNMT1-TAP localizes to both the soluble and insoluble fractions of rmitochondria. (A) Mitochondrial DNA nucleoids were isolated as described by Garrido, *et al.* (61) to determine where mtDNMT1 partitions on a sub-mitochondrial level. The soluble (free) supernatant was separated from the insoluble pellet, which contains mtDNA and mtDNA binding proteins. Using an antibody to DNMT1, mtDNMT1 was seen to localize with both soluble “S” and insoluble “P” fractions. (B) Both soluble and insoluble forms of mtDNMT1 exhibit catalytic activity *in vitro*. Values represent fluorescence readings normalized to the relative proportion of mtDNMT1 in each fraction, as determined by densitometry of the immunoblot in (A).

Figure S2: Immunoblots assessing purity of mitochondrial extracts for DNMT assays

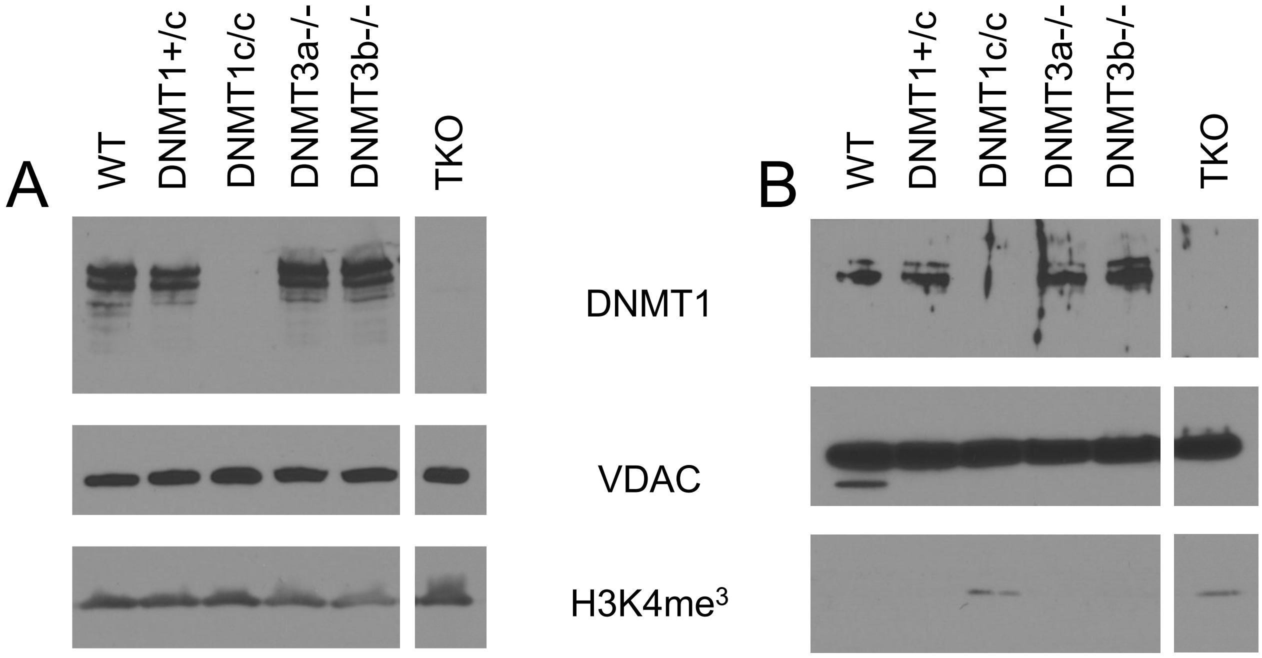

Figure S2. Immunoblots demonstrate purity of mitochondrial extracts for DNMT activity assays. Whole cell lysates (A) and purified mitochondrial lysates (B) were separated by SDS-PAGE and probed for DNMT1 and compartment-specific control antibodies to validate purity of mitochondrial enzyme preparations prior to DNMT activity assays shown in Figure 1G. DNMT1+/c, heterozygous catalytic knockout of DNMT1 (1 allele); DNMT1c/c, homozygous catalytic knockout of DNMT1 (both alleles); DNMT3a-/-, DNMT3a knockout; DNMT3b-/-, DNMT3b knockout; TKO, triple knockout, lacking all three DNMTs. *(Two lanes of the blots were excluded above as they contained material that was not used in DNMT enzyme assays in Figure 1G.)*

Figure S3: Loss of p53 induces upregulation of mtDNMT1

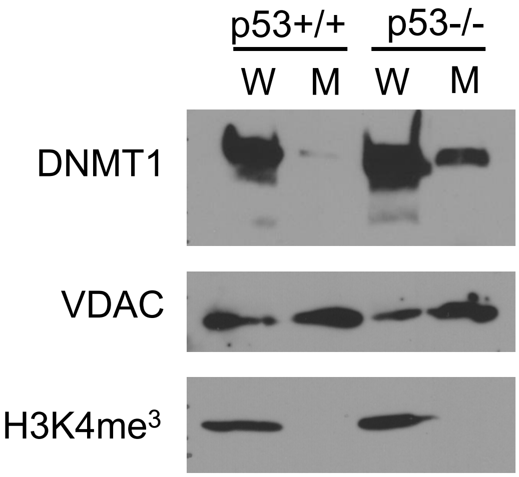

Figure S3. mtDNMT1 is upregulated by loss of p53. (A) p53+/+ and p53-/- HCT116s were fractionated into whole cell and purified mitochondrial lysates. Immunoblots probed for DNMT1 as well as compartment-specific controls show that mitochondrial DNMT1 levels increase substantially in response to p53 deletion. Total RNA isolated from these cells was used for strand-specific priming of mitochondrial cDNA to assess the effects of upregulation of mtDNMT1/loss of p53 on mitochondrial transcription in Figure 3.

Figure S4: MeDIP of mESCs

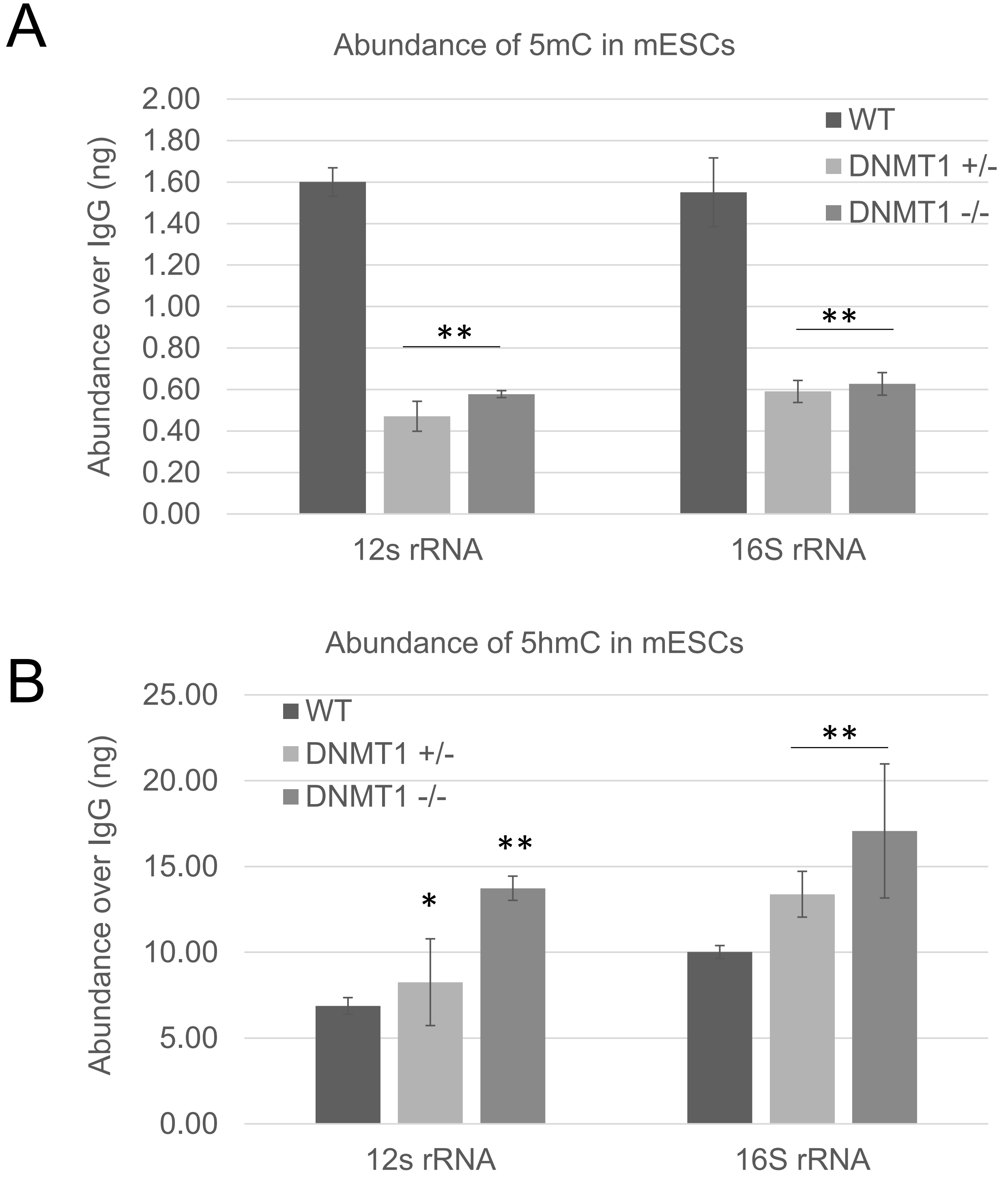

Figure S4. Loss of DNMT1 results in a reduction in 5mC and an increase in 5hmC residues in mtDNA. (A) Total gDNA from murine embryonic stem cells lacking one or both alleles of DNMT1 were subjected to MeDIP and (B) hydroxy-MeDIP analysis. Welch's two-tailed t-test, *$p < 0.05$, $p<0.01$.

Figure S5: mtDNA content analysis

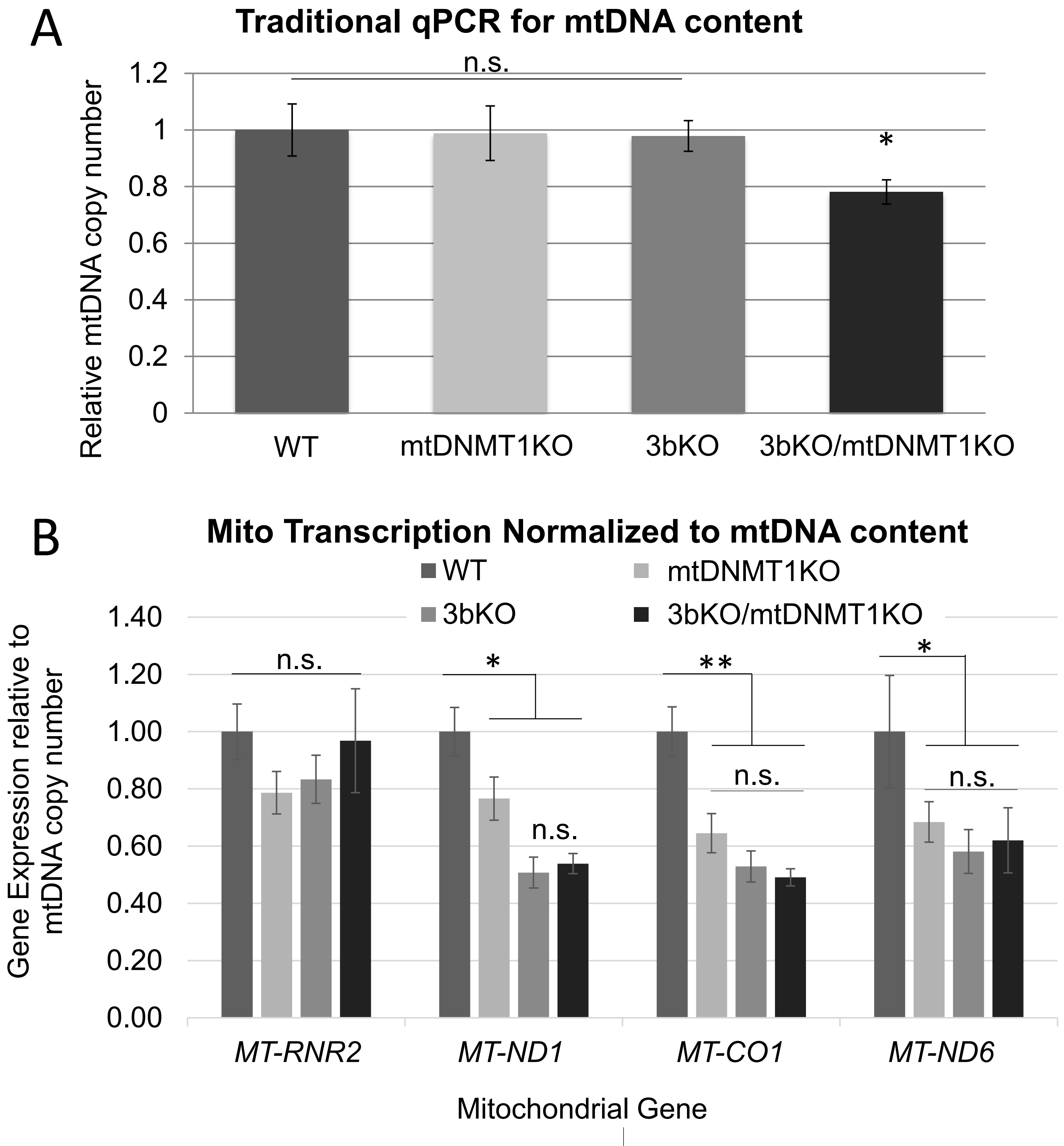

Figure S5. Loss of mtDNMT1 and/or DNMT3b results in decreased mtDNA content and decreased mitochondrial gene expression that are independent effects. (A) Traditional qPCR quantitates the reduction in mtDNA content. (B) Normalizing the transcriptional differences to the change in mtDNA content shows that there is still a substantial decrease in transcription upon loss of mtDNMT1/DNMT3b in mitochondria. Welch's two-tailed t-test, *p< 0.05, p<0.01.
